# Optical Biosensors for the Detection of Rheumatoid Arthritis (RA) Biomarkers: A Comprehensive Review

**DOI:** 10.3390/s20216289

**Published:** 2020-11-04

**Authors:** José Javier Imas, Carlos Ruiz Zamarreño, Pablo Zubiate, Lorena Sanchez-Martín, Javier Campión, Ignacio Raúl Matías

**Affiliations:** 1 Electrical, Electronics and Communications Engineering Department, Public University of Navarra, 31006 Pamplona, Spain; josejavier.imas@unavarra.es (J.J.I.); pablo.zubiate@unavarra.es (P.Z.); natxo@unavarra.es (I.R.M.); 2 Institute of Smart Cities (ISC), Public University of Navarra, 31006 Pamplona, Spain; 3 Making Genetics S.L., Plaza CEIN 5, 31110 Noáin, Spain; lsanchez@making-genetics.eu (L.S.-M.); jcampion@making-genetics.eu (J.C.)

**Keywords:** optical biosensors, rheumatoid arthritis (RA), biomarkers, miRNA, CRP

## Abstract

A comprehensive review of optical biosensors for the detection of biomarkers associated with rheumatoid arthritis (RA) is presented here, including microRNAs (miRNAs), C-reactive protein (CRP), rheumatoid factor (RF), anti-citrullinated protein antibodies (ACPA), interleukin-6 (IL-6) and histidine, which are biomarkers that enable RA detection and/or monitoring. An overview of the different optical biosensors (based on fluorescence, plasmon resonances, interferometry, surface-enhanced Raman spectroscopy (SERS) among other optical techniques) used to detect these biomarkers is given, describing their performance and main characteristics (limit of detection (LOD) and dynamic range), as well as the connection between the respective biomarker and rheumatoid arthritis. It has been observed that the relationship between the corresponding biomarker and rheumatoid arthritis tends to be obviated most of the time when explaining the mechanism of the optical biosensor, which forces the researcher to look for further information about the biomarker. This review work attempts to establish a clear association between optical sensors and rheumatoid arthritis biomarkers as well as to be an easy-to-use tool for the researchers working in this field.

## 1. Introduction

Rheumatoid arthritis (RA) is considered to be the most common type of autoimmune arthritis by the American College of Rheumatology (ACR) [1] as well as being the most common type of inflammatory arthritis in adults, affecting approximately between 0.5% and 1.0% of the adult population worldwide [2].

The inflammation that is derived from suffering rheumatoid arthritis affects the synovium, the membrane that lines and lubricates the joints by producing synovial fluid, causing synovitis, therefore provoking joint pain, stiffness and swelling [3]. The smallest joints (wrists, elbows, knees, ankles and those present in hands and feet) tend to be attacked by inflammation in RA patients, nevertheless, any joint in the body can be affected. On the other hand, although RA usually affects the joints, synovitis can expand and damage other tissues and organs; a fact that leads us to consider RA as a systemic disease [3].

Early identification of rheumatoid arthritis can affect its development, preventing joint erosion or slowing down the progression of erosive disease. It can even lead to remission after proper treatment [4]. Nevertheless, correctly identifying rheumatoid arthritis in patients cannot be considered as a closed matter. Literature is continuously revised to recover the most reliable RA disease activity measures to improve the accuracy of the diagnosis. In 2019, the ACR updated its recommendations on rheumatoid arthritis disease activity measures considering that among all the practices found in the literature, 11 fulfilled a minimum standard for regular use in most clinical settings and only 5 of them, enumerated below, were recommended [5].

1. Disease Activity Score in 28 joints (DAS28) combined with Erythrocyte Sedimentation Rate (ESR) or C-reactive protein (CRP);
2. Clinical Disease Activity Index (CDAI);
3. Simplified Disease Activity Index (SDAI);
4. Routine Assessment of Patient Index Data 3 (RAPID3);
5. Patient Activity Scale-II (PAS-II).

The previously listed RA disease activity measures employ, among other parameters, the number of swollen and tender joints out a total of 28 (DAS28, CDAI, SDAI), the patient and doctor’s global assessment of disease activity (CDAI, SDAI, only the patient in PAS-II), questionnaires for the patient (RAPID3, PAS-II), and CRP level (SDAI, DAS28-CRP) or ESR (DAS28-ESR).

Nevertheless, these measures mentioned above also have their drawbacks [6]. For example, RAPID3 and PAS-II are based on the patient’s assessment and it could be considered that they lack the reliability of the formal assessment undertaken by a doctor. In DAS28-ESR or DAS28-CRP, the use of several parameters adds complexity to the method and the fixed weighting employed for result calculation can lead to wrong estimations. Finally, CDAI is also considered as time-consuming by some researchers.

The latter measures mostly include clinical endpoints, variables that represent a study of the patient’s health and wellbeing from the patient’s perspective, such as the number of swollen and tender joints. Disease activity measures based on biomarkers are suggested here as an alternate solution to the disadvantages of RA clinical endpoints. A biomarker, also known as biological marker, is defined as an objective and quantifiable indicator of the medical condition of the patient, which can be observed from outside the patient and can be measured precisely and reproducibly [7]. It has to be stated that CRP and ESR are, indeed, biomarkers, but in the previously explained disease activity measures they were always combined with clinical endpoints.

A multi-biomarker disease activity (MBDA) test for rheumatoid arthritis was developed based on 12 biomarkers (out of 130 candidates) and a mathematical algorithm [8]. This test has given satisfactory results in measuring the disease activity in RA patients treated with rituximab [9] and in a computer-assisted management study in early RA patients [10]. However, in a study with RA patients treated with abatacept or adalimumab, no association between the MBDA test result and other previously mentioned disease activity measures (CDAI, SDAI, DAS28-CRP, RAPID3) has been found [11]. Nevertheless, this approach based on biomarkers and linked with the utilization of biosensors shows a promising path, as it has the potential for providing clinically valid data on disease activity without depending on the doctor or the patient’s assessment.

Biosensors are developed to obtain accurate data from fluids that originate in the human body, including plasma, blood or urine, among others. They basically consist of a bioreceptor, that binds the target molecule (the biomarker); and a transducer, which is intimately linked to the bioreceptor and enables a measurable response to be obtained. On the other hand, in the case of optical transducers, interrogation techniques primarily consist of light-intensity, phase, and frequency or polarization modulation induced by the bioreceptor in the presence of the biomarker. Optical biosensors are becoming increasingly important, particularly in the case of DNA and RNA biomarkers [12]. This is a direct consequence of their advantages, among which it has to be stressed their capability of a direct, real-time and in some cases label-free detection, as well as their high specificity, sensitivity, compact size and good cost-performance ratio [13].

The current review focuses on optical biosensors that enable the detection of biomarkers associated to RA. Furthermore, this review provides a clear connection between the biomarkers and RA. These connections tend to be ignored in the works that present novel optical biosensing platforms, which are focused on the detection technique and not on the medical application. 

On the other hand, biomarkers clearly connected with RA in medical literature are not associated with this particular disease. In many cases it is because some particular biomarkers cover a wide range of diseases, as happens with CRP, a general biomarker for inflammation [14,15,16]. On other occasions, the corresponding biomarker has been related to several diseases, and more attention has been paid to some of them over RA. It is the case of miR-21, linked to several types of cancer [17,18,19,20] as well as RA [21,22,23,24]. Other biomarkers are introduced as general biomarkers of disease without specifying their particular medical conditions, as is the case of let-7a. Finally, there are biomarkers that are considered important in RA medical literature, such as the rheumatoid factor (RF) [25], but not many optical biosensors have been developed in this case.

This work will be divided into three separate sections. First, the utilization of optical biosensors for the detection of microRNAs, commonly referred as miRNAs, will be described. MiRNAs are considered promising biomarkers for the detection of a wide range of diseases, including rheumatoid arthritis. Then, a second section will be focused on optical biosensors for the detection of CRP, one of the main biomarkers for RA monitoring; followed by a third section where other RA biomarkers that have also been detected by employing these techniques are mentioned. Finally, conclusions and an outlook section are also included.

Every section includes a table where the information about the different optical sensors used for the detection of the corresponding biomarker/biomarkers is summarized: Table 1 for miRNAs (Table 2 and Table 3 provide further information about the miRNAs themselves), Table 4 for CRP and Table 5 for the remaining biomarkers. The information in the tables is ordered in columns that detail the optical technique/principle that has been employed, the detected biomarker/biomarkers linked to RA, the dynamic range, the limit of detection (LOD), the specificity assay and a brief description of the sensor. Some general considerations of the data summarized in the tables are required for proper reading as detailed in the next paragraphs.

Regarding the dynamic range, in general, the linear range of the sensor will be provided, where ‘linear range’ means that, for this interval, the relationship between the response of the sensor and the concentration of the considered biomarker (usually in log scale) is linear. On some occasions, the studied range was larger and only the part where the relationship was linear has been indicated. However, in some cases, either the relationship is not linear, or the linearity has not been studied. In other sensors, other types of relationship between the sensor response and the concentration are specified. Whenever the range provided is not linear, it will be indicated.

Concerning the limit of detection (LOD) column, it includes the concentration that has received this designation in the corresponding article. In general, the LOD is defined as the lowest analyte concentration that can be detected in a sample, but not necessarily quantified, under the stated conditions of the test [26]. In some cases, the limit of quantitation (LOQ) is provided, either as a complement or a substitute for the LOD, and it will be indicated. The LOQ is the lowest analyte concentration that can be determined in a sample with acceptable precision and accuracy under the stated conditions of test [26]. In some other sensors, neither the LOD nor the LOQ are provided in the corresponding article. In these cases, the lowest concentration that has been found to be detected in the article is included and this circumstance will be explicitly mentioned.

With regard to the specificity assays, they are carried out to check that other substances that are not the desired target produce an irrelevant response of the sensor (or no response at all). In the corresponding column, the DNA chains, miRNAs, proteins, etc. that have been used for these assays are included. Other aspects of each table will be commented on in each corresponding section.

## 2. MicroRNAs or miRNAs

MicroRNAs or miRNAs are small (around 20 nucleotides in length) and non-coding RNAs (ribonucleic acids) involved in the regulation of gene expression. This regulatory function is accomplished through the RNA-induced silencing complex (RISC). MiRNA assembles into RISC, which targets the messenger RNA (mRNA, responsible for protein synthesis) specified by the miRNA, therefore reducing the expression of the gene that was codified in the mRNA. There are two possible silencing mechanisms: mRNA cleavage or translation repression, where the mechanism that takes place depends on the degree of complementarity between the miRNA and the mRNA target [27].

MiRNAs were originally discovered in *Caenorhabditis elegans* (a species of soil-dwelling nematode) [28] and they are found in most eukaryotes, including humans. The mammalian genome is reported to host around 2200 different miRNA genes, from which over 1000 correspond to the human genome. Furthermore, one third of the human genome is estimated to be regulated by miRNAs [29].

**Figure 1:**
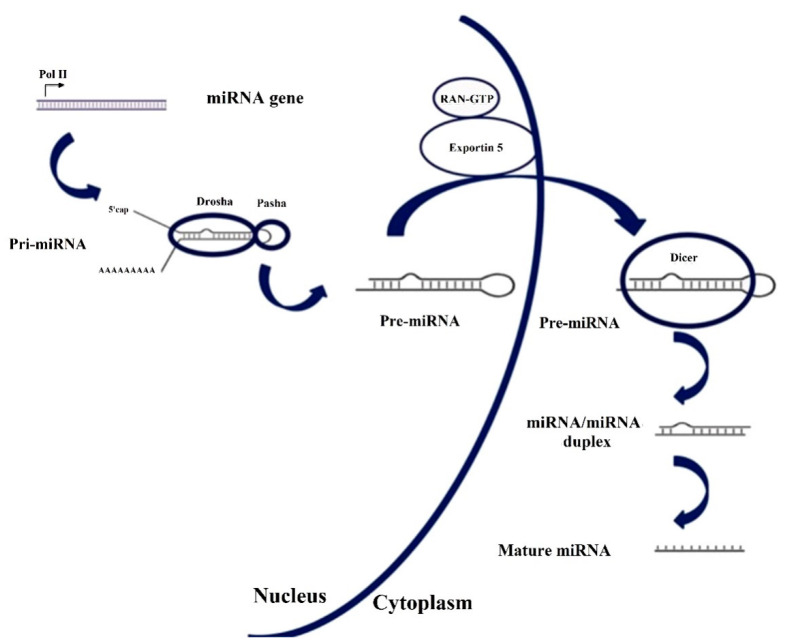
Biosynthesis pathway for miRNA. Reproduced under the terms of the Creative Commons Attribution-Non Commercial 3.0 Unported License (https://creativecommons.org/licenses/by-nc/3.0/) [29]. Copyright 2010, The Authors. Published by Avicenna Research Institute (ARI).

The genesis of miRNA involves several steps, shown in Figure 1. In the nucleus, RNA polymerase II transcribes DNA (usually referred to as miRNA genes in this process, as in Figure 1) to a primary miRNA (pri-miRNA). The pri-miRNA is processed to form a precursor stem-loop structure, called pre-miRNA. Then, the pre-miRNA is transported into the cytoplasm and cleaved by the Dicer RNAase III endonuclease to form a miRNA duplex (miRNA:miRNA*, passenger strand designated with an asterisk). The duplex unwinds and the mature miRNA, which is the one that assembles into the RISC, is obtained [27,29]. The notation miRNA and miRNA* in the miRNA duplex was originally introduced to indicate that the ‘miRNA’ was the one that generated the mature miRNA and the ‘miRNA*’ was degraded. However, it was later discovered that the miRNA* is not always degraded and can also generate mature miRNA and play a regulatory role [30].

In recent years, the implication of miRNAs in human diseases has been thoroughly studied, including cancers (ovarian, liver, bladder, colon), viral infections (hepatitis B, hepatitis C), cardiovascular disease, neurodegenerative diseases (Alzheimer, Huntington’s disease) or autoimmune diseases (rheumatoid arthritis, systemic lupus erythematosus) [29,31]. Regarding autoimmune diseases, research has been carried out in depth for rheumatoid arthritis (RA), identifying miRNAs and their connection with the disease [21,22,32,33,34,35]. Nevertheless, it has to be taken into account that in RA, several miRNAs, as well as other biomarkers, should be considered in order to provide an accurate diagnosis. Furthermore, one miRNA can be dysregulated in several diseases, that can be related, as RA and systemic lupus erythematosus; or not, as RA and different types of cancer. 

The basic operation of a biosensor for miRNA detection consists of using a single-stranded DNA sequence that is complementary to the desired target miRNA. It must be considered that there are five nucleobases (or simply, bases): adenine (A), cytosine (C), guanine (G), thymine (T) and uracil (U). A, C, G, T are present in DNA and A, C, G, U in RNA, with miRNA being a type of RNA, as it has been previously explained. Bases C and G are complementary, and A is complementary with T in DNA and U in RNA. This complementary DNA (cDNA) sequence is usually called DNA probe or capture probe. Sometimes the term hairpin probe is used due to the type of DNA that is employed. The hybridization of the DNA probe with the corresponding miRNA will be directly or indirectly measured by means of an optical technique or principle in the case of the biosensors covered in this review.

The information about the different sensors to detect miRNAs associated with RA is summarized in Table 1. The detection of each miRNA is usually performed separately. If it is not the case, it will be explained in the text. The detection of other miRNAs not related to rheumatoid arthritis is not given in Table 1. Some articles have been included not due to the low LOD or the detection range but because the corresponding technique is not commonly used for miRNA detection. In other cases, several miRNAs can be detected simultaneously or the detected miRNA cannot be easily found in optical sensors literature. 

Table 2 lists all the miRNAs linked with rheumatoid arthritis that are detected in the articles included in this review. Here, the full name of each miRNA, other names that also reference the same miRNA and the corresponding miRNA sequence [36], are included. The last two columns contain the references to the sensing platforms mentioned in this review that enable their detection (‘Ref (optical sensors)’ column) and the references in which the association of the corresponding miRNA with rheumatoid arthritis is explained (‘Ref (RA)’ column).

With respect to amplification techniques, they are commonly used in biosensors for miRNA detection, such as catalyzed hairpin assembly (CHA) [39,41] or rolling circle amplification (RCA) [43,44]. An interesting case is duplex-specific nuclease (DSN) assisted signal amplification, which has been observed in different types of optical sensors: fluorescence [38,54], plasmon resonances [61], and surface-enhanced Raman spectroscopy (SERS) [72]. Another particular case is presented in [44], where two techniques are combined: RCA and nicking enzyme amplification. The purpose of these techniques is to improve the performance of the sensor, although they increase the complexity and time of detection. For instance, in [41], CHA enables the LOD to be reduced for miR-21 detection from 9.1 nM to 47 pM (194 times) but it takes 4.5 h longer to prepare the sensor. These amplification techniques will be mentioned in the following explanations; however, they will not be described in depth, as it is not the purpose of this review and they can be consulted in the corresponding references. 

Concerning the nomenclature, the first three letters that appear in the full name of a miRNA correspond to the organism, with ‘hsa’ (from *Homo sapiens*) corresponding to humans. Lettered suffixes correspond to closely related mature sequences. There are also exceptions to the typical naming structure, such as the let-7 family, which has retained the name that was originally given [87].

Sometimes, see Figure 1, two miRNAs are derived from the same gene, which is named with the prefix ‘mir’ instead of ‘miR’. If one of the miRNAs predominates over the other, they are named miR-126 (the predominant product) and miR-126* (from the opposite arm of the precursor, designated as miRNA* in the explanation about the genesis of miRNAs). When there is no sequence that can be considered predominant, names like hsa-miR-21-5p (from the 5′ arm) and hsa-miR-21-3p (from the 3′ arm) are used [87]. Nevertheless, both notations coexist, as can be checked in Table 2.

In this review, the miRNAs are named in the text without ‘hsa’ and preferentially employing the first notation explained (predominant and non-predominant, without and with *) because it is the most common one found in the literature. However, the second one is also used in this review when required. Further explanations are given in these cases to avoid confusion. Nonetheless, as a general recommendation when searching in literature, it is always recommended to contrast the name and the sequence given with a biological database. 

Returning to Table 1, regarding the specificity assays, in most cases miRNAs differing in a few bases (1, 2 or 3) from the target miRNA are used. Sometimes they are mature miRNA and in other cases they are artificial miRNA that have been designed for these assays. In some other cases, the miRNAs used for the specificity assay are other miRNAs that have been detected in the article (cross specificity assays) or other common miRNAs. All the information about the miRNA that have been employed in specificity assays is summarized in Table 3. The miRNAs that have been detected in some works and used in specificity assays in others are included in Table 2 and their role in each article is made clear.

Among the optical sensors used for the detection of miRNAs associated with RA, the great majority of papers found in the literature are based on fluorescence or related techniques as described in the next subsection (Section 2.1). However, there are also sensors based on plasmon resonances, microring resonators, interferometry, and SERS as will be detailed in Section 2.2 and Section 2.3. This order is also followed in Table 1.

### 2.1. Fluorescence-Based Biosensors

Fluorescence sensors consist basically of using a light source at a certain wavelength (excitation wavelength) to excite a fluorophore or label and measuring the fluorescent response at another wavelength (peak wavelength) [93,94]. The difference in wavelength between the excitation (usually lower) and emission (usually higher) wavelengths is known as the Stokes shift [95].

In the case of biosensors, the sensing mechanism links the analyte concentration with the fluorophore concentration, where this relationship can be direct (the higher the concentration of the analyte, the higher the light intensity) or inverse (the higher the concentration, the lower the intensity). Fluorescence biosensors are characterized by a high sensitivity and specificity, but in order to measure the light emitted by the label precisely, the Stokes shift must be as large as possible [95]. Their drawbacks are those associated with the utilization of fluorophores, including undesired effects that affect the sensor performance such as photobleaching or self-quenching.

In the listed sensors for miRNA detection, the peak wavelengths tend to be in the range 520–530 nm, and the fluorophores that have been employed include: 5(6)-carboxyfluorescein (FAM) [38,39,43,44,50,51,54,59], SYBR Green I [45,48,52,58], Cy5 [46,56], poly(3-alkoxy-4-methylthiophene) (PT) [37], TAMRA [41,54], YOYO-1 [42], Atto 550 [56], FITC [56], Eosin Y [40], Rhod-5N [47], Cy3 [53], Oyster 556 [57], and Oyster 656 [57]. The fluorophores whose chemical composition has not been specified correspond to compounds that are widely known. More information can be consulted in [96] except in the case of Rhod-5N, Oyster 556 and Oyster 656, which are commercial names. The same criterion is applied for the rest of the fluorophores that are mentioned in the current review. In [49,55], quantum dots (QDs), a kind of fluorescent semiconductor nanocrystals, are utilized as fluorescent labels instead of fluorophores. There are sensors in which several fluorophores [56] or QDs [49] are employed with different miRNAs in order to enable their independent detection, and in [57], both fluorophores are used in every miRNA. Finally, fluorophores are also utilized in SERS sensors [97], which will be explained in Section 2.3.

**Figure 2:**
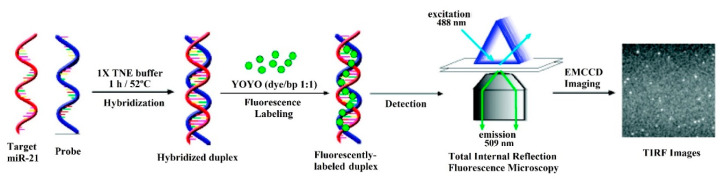
Schematic illustration of the hybridization-based total internal reflection fluorescence microscopy (TIRFM) assay for the detection of single miR-21 molecules in solution in which the fluorophore YOYO-1 is used. Reproduced with permission from [42]. Copyright 2010 American Chemical Society.

However, in some cases, the mechanism of the sensor is more complex and involves the use of a certain fluorescence technique. This is the case of total internal reflection fluorescence microscopy (TIRFM), utilized in [42]. In TIRFM, a laser beam experiments total internal reflection, generating an evanescent field layer that enables single molecule detection (SMD) of the fluorescent molecules (see Figure 2). In [42], the hybridization of the DNA probes and the miRNA is monitored with an electron-multiplying charge-coupled device (EMCCD) coupled to the TIRFM system.

Another fluorescence-based technique is fluorescence anisotropy (FA) [41], phenomenon in which the light emitted by a fluorophore changes depending on the axis of polarization used for the measurement. In [46], the corresponding technique is called photonic crystal-enhanced fluorescence (PCEF), due to the use of a low index SiO_2_ grating on top of a silicon substrate and overcoated with a high index TiO_2_ layer. The periodic arrangement of the high and low index layers results in a narrow band resonance peak and amplifies the output of surface-based fluorescent assays.

Regarding the materials, graphene oxide (GO) is present in an important part (more than half) of the sensors for fluorescence-based miRNA detection included in this review, either as GO in [39,41,43,44,45,48,51,52,53,55,56,59], or as reduced graphene oxide (rGO) in [40,58]. A particular case for GO is shown in [43], where it is used is in the shape of nanoplates. The electrical, mechanical, thermal and chemical properties of graphene have led to its use in optical sensors based on various interrogation schemes (resonance, interferometry, …) including fluorescence [98]. However, further research in micro- and nanostructured materials is considered critical to improve current optical biosensors performance [99,100]. 

It can be checked in [101] that graphene is a common material in biosensors for miRNA detection, not only optical, but also electrochemical. There are two main reasons for the utilization of GO in the case of fluorescent sensors for miRNA detection [101]. Firstly, GO is capable of quenching the light emitted by a fluorophore in close proximity to its surface. In the second place, GO has a high affinity towards single-stranded (ss) nucleic acids (DNA or RNA), but not towards double-stranded (ds) nucleic acids (for example, a DNA hybridized with the corresponding miRNA). 

**Figure 3:**
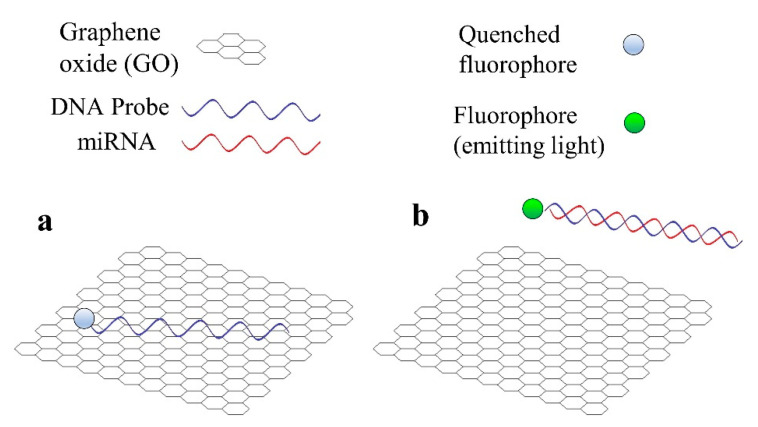
Basic operation principle of a fluorescent sensor for miRNA detection that employs graphene oxide (GO). (**a**) DNA probe is adsorbed by GO and the fluorophore is quenched. (**b**) miRNA hybridizes with DNA probe and both desorb from GO, the fluorophore emits light.

Therefore, the basic operation of a fluorescent sensor that employs GO is the following: a fluorophore (or a QD) is attached to one end of the DNA probe, which will be adsorbed by the GO, thus the fluorescence is quenched (Figure 3a). This situation will only change in the presence of the target miRNA, which will hybridize with the DNA probe; causing the resulting DNA-RNA hybrid to desorb from the GO surface. The fluorophore, no longer close to the GO surface, will be able to emit light, enabling us to know that the detection has taken place (Figure 3b). This basic operation principle is usually combined with more complex amplification strategies, as happens in all the previously mentioned sensors.

Fluorescence-based sensors for the detection of miR-21, being the most-numerous, are explained in the first place (Section 2.1.1). Then, fluorescence biosensors for let-7a detection are described (Section 2.1.2) and finally, those that detect other miRNAs are detailed in Section 2.1.3. This order is also followed in the fluorescence section of Table 1, listing the sensors in order of decreasing LOD.

#### 2.1.1. MiR-21

MiR-21 has been identified as a biomarker for bladder and prostate cancer [17], breast cancer [18], non-small cell lung cancer [19], or pancreatic cancer [20], which justifies the interest in developing sensors for the detection of this miRNA. 

In the case of RA, miR-21 may be related to the imbalance of proinflammatory T helper 17 cells (Th17) and anti-inflammatory regulatory T cells (Treg), which contributes to RA development [23]. Th17 cells produce interleukin-17 (IL-17) and are relevant contributors of inflammatory responses in RA [102], whereas Treg cells are a specific subpopulation of T cells that behave as suppressors of immune response [103]. On the one hand, miR-21 levels are decreased in peripheral blood mononuclear cells (PBMCs) in RA patients compared with healthy controls, which is associated with an increase and activation of STAT3 (signal transducer and activator of transcription 3), a transcription factor that is involved in the differentiation of Th17 cells [21]. On the other hand, low miR-21 levels in RA patients are connected with reduced counts of circulating Treg [23], while high levels of miR-21 in the synovial fluid promote the accumulation of memory Treg cells linked to antiapoptotic processes [24].

Before describing the fluorescence sensors for miR-21 detection, another aspect will be commented on. In general, the media in which miRNAs are detected using optical biosensors are different types of buffer. However, in some cases miR-21 detection is also assessed in tumor cell lines, such as MCF-7, a type of breast cancer cell line [39,42,43,47,49], due to the connection of miR-21 with this cancer [18]. These assays justify the employment of the corresponding biosensor for breast cancer diagnosis. This type of assays (for other tissues and types of cancer) are quite common, not only for miR-21 but also for the rest of the miRNAs and types of optical sensors included in Section 2. Nevertheless, no more attention will be paid to them, as they are not under the scope of the current review.

On the other hand, on very few occasions, these sensors are used to detect miRNAs in human plasma or sera. Although in these cases plasma or sera usually come from cancer patients, and the purpose is to demonstrate the feasibility of the diagnosis of the corresponding cancer (not RA); this information will be remarked upon, because an analogous assay could be carried out with plasma or serum from RA patients for rheumatoid arthritis diagnosis. 

Apart from the fluorescent labels explained in the introduction of Section 2.1, there are also other elements that have been employed as part of the sensing strategies for miR-21 detection. For instance, in [38], gold nanoparticles (Au NPs) are used to quench the light from the fluorophore in the absence of miR-21 and are also responsible for the colorimetric response due to their surface plasmons. 

Moreover, magnetic particles and CdSe nanocrystals are employed in a 3-step procedure in [47]. In the first step, magnetic particles coupled to DNA probes are suspended in a solution containing a miRNA, that will only hybridize with the DNA probe if it is miR-21. The particles are collected with a magnet and introduced in a second solution, which contains CdSe nanocrystals (CdSe NCs) coupled to another probe. This probe will only hybridize with the initial DNA probe if the miR-21 has hybridized in the first place. After washing several times, a third solution containing Rhod-5N molecules is added. Only if both hybridizations have taken place (that is, only if miR-21 has been detected), will there be CdSe NCs in this third step. In that case, Cd2+ ions are released from each nanocrystal, turning on the fluorescence of Rhod-5N molecules. 

Magnetic silicon microspheres (a type of magnetic nanoparticles, MNPs), similar to the magnetic particles in [47], are used in [40]. In this work, rGO (whose properties, explained in Section 2.1, play an important role) and DNA probes attached to the MNPs are introduced in an aqueous solution and a magnetic field is applied. In the absence of miR-21, the MNP-DNA probes are adsorbed by the rGO and the magnetic field will separate both from the solution. In the presence of miR-21, the MNP-DNA-miR-21 hybrids will be desorbed from the rGO and only the hybrids will be separated from the solution after applying the magnetic field, while the rGO will remain in the solution. After this step, the fluorophore Eosin Y is introduced in the solution. Only if miR-21 has been detected, the fluorescence of Eosin Y will be quenched by the remaining rGO, so the fluorescence quenching is related to the detected miR-21 concentration. It is important to remark that this sensor enabled measurement of concentrations of 1, 10 and 40 nM of miR-21 spiked in 100 times diluted human serum, with a recovery rate (the recovered concentration divided by the added concentration and expressed in percentage) of 95.52–120.3%.

**Figure 4:**
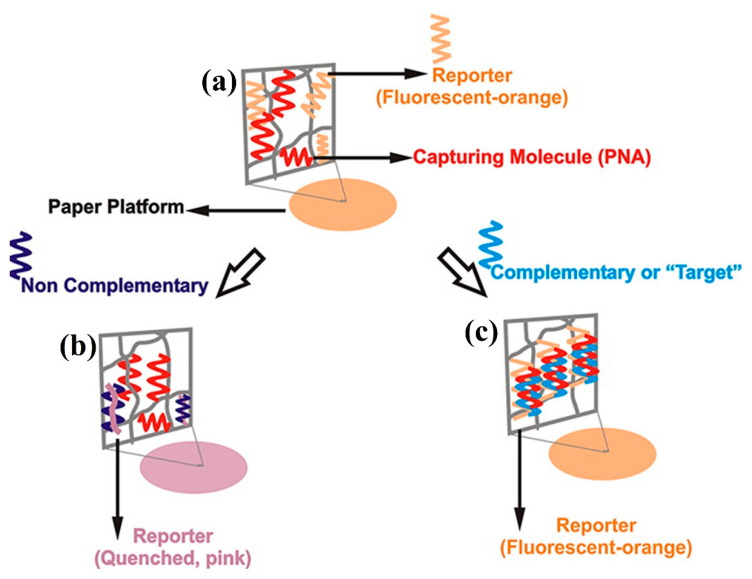
(**a**) Paper platform for the detection of miR-21. (**b**) Case where the target is not detected, the fluorescence is quenched and the color changes from orange to purple. (**c**) Case where the target miRNA is detected and orange fluorescence is maintained. Reproduced with permission [37]. Copyright 2012 American Chemical Society.

Although graphene oxide is the most common used material in fluorescence based biosensors for miRNA detection, other materials are also utilized. A paper-based biosensing platform for detection by the naked eye of miR-21 is described in [37], (see Figure 4). To be precise, it is a poly(vinylidene fluoride) (PDVF) impregnated thin paper that uses PT as luminescent reporter. If miR-21 hybridizes with the DNA probe (named capturing molecule in Figure 4), the orange fluorescence signal from PT is maintained (Figure 4c). In any other case, the fluorescence is quenched and a color transition from orange to purple is observed (Figure 4b).

Another particular case is presented in [38], where not only fluorescence, but also colorimetry (ratio of the absorption at λ = 620 nm and λ = 520 nm, as a measurement of the change from red to blue) is employed. This dual mode sensor is based on FAM labelled hairpin probes combined with Au NPs and DSN-assisted signal amplification. Here, Au NPs act as fluorescence quenchers in the absence of miR-21. In contrast, in the presence of miR-21, both FAM and Au NPs are released producing the fluorescent and the colorimetric response, respectively. With the first method (fluorescence), a linear range from 50 pM to 1 nM with a LOD of 50 pM is obtained. The second one (absorbance) provides a LOD of 300 pM and a linear response in the range 300 pM–8 nM.

The LOD is in the order of pM for most of the listed miR-21 fluorescence sensors. However, in [47,48] the LOD is in the fM range. In [47], an amplification method based on cation exchange (named CXFluoAmp) combined with CdSe nanocrystals and Rhod-5N molecules allows a LOD of 35 fM to be achieved with a dynamic range of slightly more than 7 decades (100 fM–5 μM), which is the best dynamic range among the sensors for miRNA detection included in this review. In [48], isothermal exponential amplification is used with graphene oxide and SYBR Green I, and the LOD is 10 times lower (3 fM), with a linear range from 10 fM to 10 pM. Here, the specificity is tested with no miRNA (control), miR-210-3p and miR-214; in all the cases with a response between 2.5 and 4.5 lower than that corresponding to the same concentration of miR-21.

**Figure 5:**
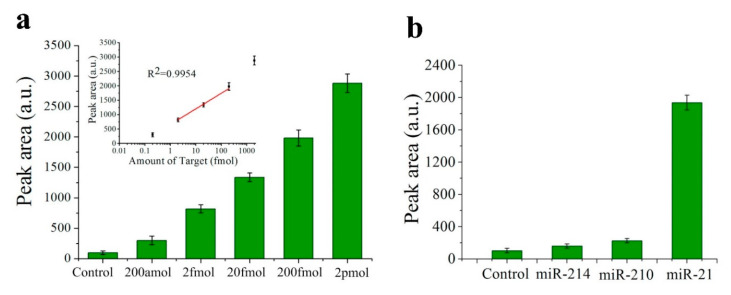
(**a**) Histograms of the peak areas on the test lines that correspond to a negative control and miR-21 in different concentrations. Inset shows the calibration plot of the peak area of the test line versus miR-21 concentration. (**b**) Histograms of the peak areas on the test lines in a specificity assay: negative control, miR-214, miR-210-3p (indicated as miR-210) and miR-21. (**a**,**b**). Reprinted [49], Copyright 2017, with permission from Elsevier.

Nevertheless, the lowest LOD among the fluorescence sensors for miRNA detection is found in [49], with a value of only 200 aM for miR-21 (see Figure 5a). In this case, the biosensor consists of a QD-605 (emission peak at 605 nm) labelled strip that employs target-recycled non-enzymatic amplification. The strip has two lines: the control line, whose fluorescence must always be visualized and confirms the validity of the strip; and the test line, whose fluorescent area (called “peak area”) will increase with miR-21 concentration. The dynamic range goes from 200 aM to 2 pM, being linear in the 2 fM–200 fM interval (see Figure 5a). In this work, specificity is also tested with no miRNA (control), miR-210-3p and miR-214 and in all the cases the fluorescence is more than 5 times lower than that corresponding to the same concentration of miR-21 (see Figure 5b).

#### 2.1.2. Let-7a

In previous literature, attention has been paid to let-7a not because its correlation with rheumatoid arthritis, but mainly due to its suitability for specificity assays. Let-7a is part of the let-7 gene family, which includes several miRNAs that differentiate in only a few bases: let-7a, let-7b (2), let-7c-5p (1), let-7d (2), let-7e (1), let-7f (1), let-7g (2) and let-7i (4). The number between parentheses is the number of bases in which the corresponding miRNA differs from let-7a (see Table 2 and Table 3 for more detailed information about the corresponding sequences). In this sense, an assay in which the corresponding sensor, which has been able to detect let-7a, does not produce a significant response in the presence of other miRNAs from the let-7 family is considered to demonstrate the specificity of the sensor.

There are articles in the literature where a ‘main’ miRNA is detected and its LOD studied. Then, let-7a (or sometimes other miRNAs linked to RA) is detected but the focus is only on the specificity assay without paying attention to its LOD. These types of article have been avoided in this review, unless the ‘main’ miRNA whose LOD is assessed is connected with RA too [57,58] or the article is relevant for other reasons.

Regarding its connection with RA, in [73] the expression of let-7a is studied in monocytes from anti-citrullinated protein antibodies (ACPA)-positive RA patients, finding that ACPA could suppress let-7a expression levels in these cells. The reduced level of let-7a could increase the expression of Ras proteins (encoded by Ras genes, where Ras stands for retrovirus-associated DNA sequences), which contribute to the destruction of the cartilage and bone in RA. On the other hand, let-7a, among other miRNAs, is significantly upregulated in the differentiation of T cells that produce interleukin-17 (IL-17), an important factor in RA pathogenesis [74]. These T cells that produce IL-17 include the previously mentioned Th17 cells [102].

In [50], let-7a is detected using carbon nanoparticles and DNA probes labelled with FAM, achieving a LOD of 3.5 nM. In this case, discrimination between let-7a and other miRNAs from the let-7 family (let-7c-5p, let-7e, let-7f) is undertaken based on the melting temperature of the DNA probe-miRNA hybrids. The melting temperature (Tm) is the temperature at which one half of the hybrids are denatured, that is, only the other half of them remains hybridized. The higher the homology (related to the number and length of the sequences of bases that are complementary), the higher the Tm [104]. In this sensor, the DNA probe is obviously complementary to let-7a and, therefore, the highest Tm corresponds to the DNA probe-let-7a hybrid. In consequence, if the temperature is increased in this sensor, there will be a certain point where the hybridization (and the associated fluorescence due to the operation of the sensor) will be relevant only if the miRNA corresponds to let-7a, enabling it to be differentiated from the others.

DNA probes labelled with FAM are also employed in [51] but in this case they are combined with a hybridization chain reaction (HCR) coupled with a GO surface in order to improve the LOD. The lowest concentration of let-7a detected is 1 pM and the specificity is studied using let-7b, let-7e, let-7f, let-7g and let-7i (concentration of 3 nM). The worst case corresponds to let-7f, with a fluorescence lower than the 40% of the fluorescence that corresponds to the same concentration of let-7a. For the rest of the miRNAs, the fluorescence is less than the 30% of the response associated to let-7a and even less than 10% for let-7g and let-7i.

A similar procedure to that described in [48], using graphene oxide and SYBR Green I, is explained in [52], but in this case for the detection of let-7a instead of miR-21. Circular exponential amplification is utilized instead of isothermal exponential amplification to improve the sensor performance. The LOD achieved in this case for let-7a is 10.8 fM (slightly higher than the 3 fM achieved for miR-21 in [48]) with a linear range from 60 fM to 12 pM. The specificity is assessed with let-7b, let-7c-5p and miR-21 (concentration equal to 0.12 nM) and for all of them the fluorescence is between 3 and 5 times lower than that for the same concentration of let-7a.

**Figure 6:**
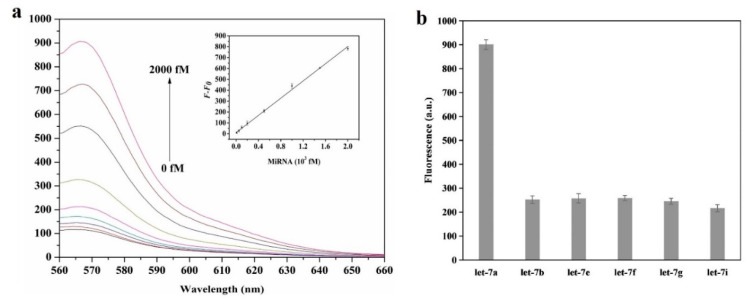
(**a**) Fluorescence spectra of the HCR/GO biosensor in the presence of different concentrations of let-7a (from bottom to top 0, 10 fM, 50 fM, 100 fM, 200 fM, 1 pM, 1.5 pM, 2 pM). Inset: linear relationship between the fluorescence intensity change (F–F_0_) and let-7a concentration. (**b**) Specificity assay with let-7b, let-7e, let-7f, let-7g, let-7i (concentration 2 pM). (**a**,**b**) Reprinted [53], Copyright 2018, with permission from Elsevier.

In [53], let-7a is detected with a platform that employs graphene oxide, helicase amplification of hybridization chain reaction (HCR) and DNA probes labelled with Cy3. The achieved LOD has a value of 4.2 fM and the linear range covers the 10 fM–2 pM region (Figure 6a). This LOD is the lowest for let-7a among the sensors included in this review, although it is quite far from the best values (200 aM [49] for fluorescence-based sensors and 10 aM [63] considering all types of optical sensor for miRNA detection). The specificity has been studied with 5 miRNAs from the let-7 family: let-7b, let-7e, let-7f, let-7g and let-7i (concentration 2 pM). In all the cases, the measured fluorescence is around 3.5 times lower than that obtained for the same concentration of let-7a (Figure 6b).

#### 2.1.3. Other miRNAs

Additional fluorescence-based optical biosensors for the detection of RA-linked miRNAs different from miR-21 and let-7a are also listed in Table 1, although the latter also appear sometimes in these works. For example, miR-155 (also an important biomarker for different types of tumor, including breast cancer [105]) and miR-21, both of them linked with RA, are detected in [55]. In this case, an elevated expression of miR-155 in peripheral blood mononuclear cells (PBMCs) is associated with RA [84]. MiR-155 is also upregulated in synovial tissue, synovial fibroblasts, synovium macrophages, whole blood; and down regulated in plasma and serum of RA patients [21].

**Figure 7:**
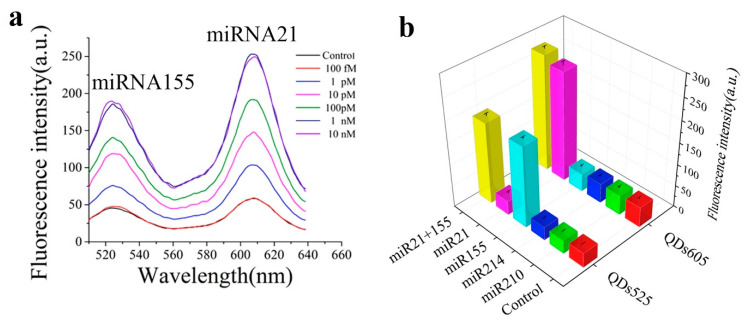
(**a**) Fluorescence spectra of samples with different concentrations of miR-21 and miR-155 (control represents the sample without any miRNA) (**b**) Specificity assay with no miRNA (Control), miR-210-3p (indicated as miR-210) and miR-214 (concentration 100 pM). (**a**,**b**) Reprinted [55], Copyright 2017, with permission from Elsevier.

The biosensor proposed in [55] consists in a nano-photon switch that employs quantum dots (QDs) and GO, producing a fluorescence resonance energy transfer (FRET). Since QD-525 and QD-605 (already seen in [49]) have been used for miR-155 and miR-21 respectively, both miRNAs can be individually detected; the response of each miRNA being associated with the respective fluorescence peak wavelength (see Figure 7a). For both miRNAs, the LOD achieved is 1 pM with a linear range from 1 pM to 1 nM. Specificity has been studied with no miRNA (blank), miR-210-3p and miR-214 (Figure 7b); which produced no relevant response.

**Figure 8:**
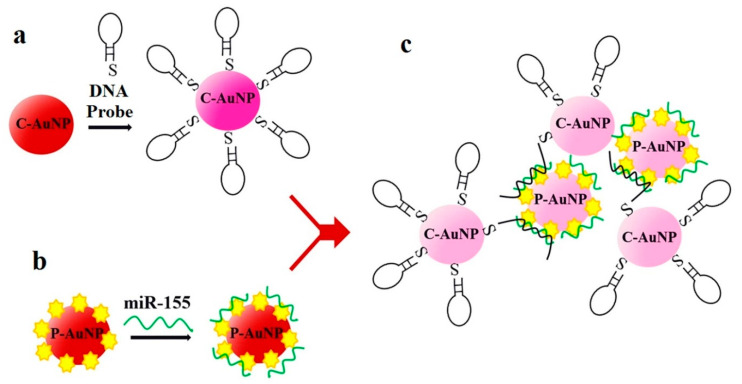
Sensor operation based on Au NPs for miR-155 detection. (**a**) Citrate-capped Au NPs (C-Au NPs) and DNA probes binding (**b**) PEI-capped Au NPs (Au NPs) and miR-155 binding. (**c**) MiR-155 detection based on the color change from red to pinkish/purple. (**a**–**c**) Reproduced under the terms of the Creative Commons Attribution 4.0 International License (https://creativecommons.org/licenses/by/4.0/) [60]. Copyright 2018, The Authors. Published by Scientific reports.

Another sensor for miR-155 detection is found in [60], although in this case it is not based on fluorescence but on absorbance, an optical detection technique previously mentioned in [38] for the detection of miR-21. In [60], DNA probes bind to negatively charged citrate-capped Au nanoparticles (C-Au NPs) in the first place, see Figure 8a. Then, the target miR-155 is electrostatically adsorbed onto the positively charged Au NPs polyethylenimine(PEI)-capped Au NPs (P-Au NPs), see Figure 8b. When both types of Au NP are mixed and the hybridization takes place, the solution color changes from red to pinkish/purple due to the coupling of the Au NPs’ surface plasmons, as it can be observed in Figure 8c. This color change depends on the miR-155 concentrations and is quantified through the ratio of the absorption at λ = 530 nm and λ = 750 nm. With this sensing strategy, the LOD achieved is 100 aM (second best LOD for miRNA detection among the sensors of this review, only beaten by [63]), with a linear range over 3 decades (100 aM–100 fM).

Another miRNA that is related to RA is miR-141, detected with a sensor that uses beta nickel hydroxide, β-Ni(OH)2, and DSN amplification [54]. It has been recently reported that miR-141 is downregulated in synovial fibroblasts (SF) from RA patients and that, combined with forkhead box protein C1 (FoxC1), has a role in RA pathogenesis by influencing inflammation and SF proliferation [83]. β-Ni(OH)2 has similar properties to those explained for graphene (quenching ability and different affinity for ssDNA and dsDNA) that enable the sensor operation. In this work [54], miR-141 and miR-21 are separately detected in the same assay thanks to the use of two fluorophores (FAM and TAMRA) although the dynamic range (1 pM–5 nM) and LOD (1 pM) are only studied for miR-141. The specificity is also assessed for miR-141 with a single base mismatched miRNA (response more than 3 times lower), miR-21, miR-200b and miR-429 (irrelevant response). 

In [56], the sensing platform is a fluorometric system that uses graphene oxide and rolling circle amplification (RCA). The LOD is only studied for miR-21, with a value of 0.4 pM. However, apart from miR-21, this sensor is employed to detect miR-16, miR-31 and miR-155 in a concentration of 10 nM as well as study the cross-specificity with the 4 miRNAs, obtaining in all the assays appreciable hybridization only between the target miRNA and the corresponding DNA probe. Here, the main interest of this work is not the LOD but the fact that these 4 miRNAs are all related to RA and the good specificity results. 

There are 2 miRNAs out of the 4 that appear in [56] whose relationship with RA has not been previously explained. MiR-16 is upregulated in PBMCs of patients with RA [21,22], and its level is low in the sera of early rheumatoid arthritis patients in comparison with established RA patients [79]. In the case of miR-31, it is overexpressed in RA patients, reducing the differentiation of mesenchymal stromal cells (MSC) into osteoblasts and adipocytes [22].

A platform with 5 lasers (green, blue, infrared and two red) for the quantitation of miRNAs is presented in [57]. In this sensor, two LNA (locked nucleic acid)-DNA probes labelled with fluorophores (Oyster 556 and Oyster 656) are used to capture each of the studied miRNAs. The characterization of the sensor is performed with miR-9 spiked into a complex RNA background (*Escherichia coli* total RNA), obtaining a dynamic range from 500 fM to 300 pM and a LOQ of 500 fM. In [77], it was concluded that miR-9 was significantly downregulated in the serum of patients with RA and peripheral neuropathy when compared with RA. MiR-9 serum levels were also reported to be low in RA patients compared to controls in a study with Chinese patients (5 RA patients and 5 controls) [78]. The specificity of the sensor is assessed with let-7a (neither the LOQ nor the dynamic range are studied in this case), using let-7c-5p (that differs in 1 base from let-7a), let-7b (2 bases), and let-7d (2 bases) as controls, with good results. The sensor response is more than 3 times lower in the case of let-7c-5p compared to that of let-7a and irrelevant in the case of let-7b and let-7d (more than 10 times lower).

The main interest of this work [57] relies on the tests performed to detect 45 miRNAs in 16 different tissues. Among the 36 miRNAs that are finally detected in these tissues, it can be consulted in the accompanying references their connection with RA: let-7a, miR-9, miR-16, miR-24, miR-126, miR-141, miR-335 (these miRNAs are detected in other works included in this review and their link with RA is explained with them), miR-22 [21], miR-25 [33], miR-28 [35], miR-30a* (miR-30a-3p) [106], miR-100 [21], miR-103 [33], miR-124a [107], miR-132 [108,109], miR-140 [22], miR-142-3p [21], miR-143 [89,90], miR-145 [90], miR-152-3p [21] (it is not clear if this reference mentions miR-152-3p or miR-152-5p), miR-210-3p [91,92] (it is not clear if these references mention miR-210-3p or miR-210-5p), and miR-221 [85,110]. Except for the first seven listed miRNAs, these miRNAs are not included in Table 2 as they have been not considered relevant enough. Their corresponding sequences can be found in [36] using the name that has been provided and the prefix ‘hsa’.

High levels of miR-126 have also been detected in RA plasma relative to human control plasma [35]. In [59], a method employing graphene oxide, DNA probes with FAM and site specific cleavage with RsaI endonuclease allows the detection of cDNA miR-126 (complementary sequence to miR-126). It must be said that the complementary sequence is generally used to detect the corresponding miRNA while in this case it is done the other way round. In this case, the achieved LOD is ~3 fM, with a linear range from 20 fM to 100 pM.

The final miRNA explained in this subsection that plays a role in RA is miR-125a. It has been identified as a plasma biomarker in rheumatoid arthritis in [35]. It is worth mentioning that miR-125a does not correlate with the presence of CRP, ACPA, RF or with DAS28; which means that it is not a mere indicator of general inflammation and it could be an independent biomarker, alternative to autoantibodies and disease activity biomarkers. MiR-125a is detected in [58] based on rGO-assisted rolling circle amplification (RCA) and the use of SYBR Green I. The LOD achieved for this miRNA is 10.3 fM, with a linear range of 4 decades (10 fM–100 pM). Let-7a is also detected (100 fM) with this sensor using the corresponding DNA probe, although neither the LOD nor the dynamic range are studied in depth. Specificity assays are carried out for both miR-125a and let-7a, where it is qualitatively shown that the sensor is specific for miR-125a (employing artificial one and two base mismatched miRNAs) and let-7a (let-7b, let-7c-5p and let-7d; that only differ from let-7a in one or two bases). In addition to this, for let-7a (100 fM) the interference of different concentrations of let-7c-5p (1 fM–10 pM) has been studied, and does not affect appreciably the sensor response.

### 2.2. Resonance-Based Biosensors

Other optical sensors for miRNA detection are resonance-based biosensors that rely on light modifications by means of the utilization of different materials covering the waveguide. These optical sensors work as refractometers [111], that is, they are able to detect small changes in the refractive index at the sensor surface, which are correlated with the analyte-binding interaction in the case of biosensors. In particular, optical fiber sensors based on resonances are considered one of the prominent technologies for biosensing applications [95,112]. 

Depending of the dielectric properties of the waveguide and the covering, different resonances take place. Surface plasmon resonance (SPR) occurs when the real part of the permittivity of the material used as a covering is negative and higher in magnitude than its own imaginary part and the real part of the permittivity of the waveguide [113]. SPR imaging, also known as SPRi, simply differentiates from conventional SPR in incorporating a CCD (charge-coupled device) camera that allows sensorgrams and SPR images to be recorded at the same time [114]. SPR stands out for having high sensitivity, good cost-performance ratio and enabling direct and real-time monitoring of the analyte binding [115,116].

In the presence of the analyte of interest, the refractive index in the region close to the biosensor surface changes, thus modifying the characteristics of the light coupled to the surface plasmons, such as the resonant wavelength, the intensity or the phase. By monitoring one of these variables, the concentration of the analyte can be obtained [117].

In [61], a SPR sensor with an Au and reduced graphene oxide (rGO) film and employing duplex specific nuclease (DSN) for signal amplification has been used for detecting miR-21 and let-7b. This device achieves a LOD of 3 fM and a dynamic range of 4 decades (10 fM–100 pM) for miR-21, whereas let-7b is detected in a concentration of 10 fM (its LOD and dynamic range are not studied) and its specificity is assessed with blank (no miRNA), let-7a, let-7c-5p and let-7e with good results (in all the cases the sensor response is more than 4 times lower than that of let-7b). 

This biosensor [61] was used to determine miR-21 levels in 13 serum samples ranging from 70 to 3400 ng/L, achieving good results. It was also employed to assess miR-21 levels in 104 clinical serum samples (diluted in buffer) from patients with different types of cancer (liver cancer, colorectal cancer, gastric cancer, lung cancer and breast cancer) as well as 20 samples from healthy individuals. Furthermore, tests were done with known concentrations (between 330 pM and 780 pM) of miR-21 and let-7b spiked into blood samples, and the recovery rates were in the range from 94.1% to 107.3%.

Although let-7b is not studied as in depth as miR-21 in [61], attention has been paid to let-7b because it is the first (and only) work included in this review where let-7b, which is also connected with RA, is treated as the target miRNA. Let-7b is usually employed in specificity assays of let-7a, such as in [51,52], or of other miRNAs. Concerning RA, let-7b contributes to arthritic joint inflammation through a mechanism that is dependent on the transformation of naive myeloid cells into M1 macrophages [75].

SPRi sensor based on Au islands and orthogonal signal amplification for the detection of miR-15a is presented in [62]. This device exhibits a LOD of 0.56 fM and a dynamic range of 5 fM–0.5 nM. This biosensor showed a recovery between 98.6% and 104.9% for four concentrations in the range 7.5 fM–7.5 nM of miR-15a spiked into 10% diluted commercial normal human sera. Furthermore, it was also tested with 40% diluted colorectal cancer patients and healthy serum with miR-21 concentrations in the fM range. Apart from being associated to colorectal cancer [118], regarding RA, miR-15a is reported to be downregulated in arthritic synovial tissue [21]. 

**Figure 9:**
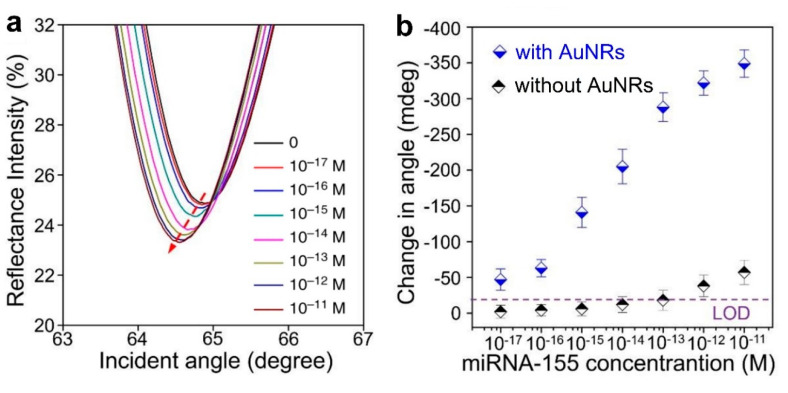
(**a**) Surface plasmon resonance (SPR) spectra with miR-155 concentrations ranging from 10^−17^ to 10^−11^ M obtained using gold nanorod (Au NR) amplification. The arrow denotes the shift in the SPR angle. (**b**) Relationship between the SPR angle and miR-155 concentration using DNA probes with and without Au NRs. (**a**,**b**) Adapted under the terms of the Creative Commons Attribution 4.0 International License (https://creativecommons.org/licenses/by/4.0/) [63]. Copyright 2019, The Authors. Published by Nature Communications.

The LOD is lowered by 2 orders of magnitude in [63], compared to previous work. Here, both miR-21 and miR-155 are detected (in separate assays) with a LOD of 10 aM and detection over a range of 6 decades (10 aM–10 pM), see Figure 9a for miR-155 (the same results are also achieved for miR-21). This label-free SPR sensor uses DNA probes with gold nanorods (Au NRs, whose importance can be appreciated in Figure 9b) and antimonene, a material similar to graphene but with better stability and hydrophilicity. It has to be remarked that this sensor possesses the lowest LOD among the works for miRNA detection included in this review (one decade lower than the second best one [60]), that this LOD is achieved for two different miRNAs (miR-21 and miR-155), and that it is also one of the best sensors in terms of the dynamic range (6 decades), only below [47,72].

Localized surface plasmon resonance (LSPR) is generally considered a particular type of SPR. The main difference is that in the case of LSPR the plasmons oscillate locally to the nanostructure instead of along the metal–dielectric interface [119]. In the case of LSPRs, the electromagnetic field intensity falls in a much shorter distance compared to SPRs, causing an enhancement of the electrical field around the nanostructure, making LSPR highly sensitive to small molecules [120]. LSPRs have already appeared in this review, as they are responsible for the colorimetric response associated to Au nanoparticles in [38,60], a phenomenon that is detailed in [121].

In [64], a label-free sensor based on LSPR with gold nanoprisms permits the detection of miR-21 with a LOD between 23 and 35 fM depending of the media (phosphate-buffered saline buffer, 40% diluted human plasma and 40% diluted bovine plasma from lower to higher LOD). The dynamic range of this biosensor in 40% diluted human serum is from 10 pM to 100 nM. This device was tested with plasma from 6 pancreatic cancer patients (for which miR-21 is also a biomarker [20]) and 6 healthy controls, with and without performing RNA extraction techniques, working with concentrations in the range of 100 ng/L.

Another type of resonance is the lossy mode resonance (LMR) sensor. LMR occurs when the real part of the permittivity of the material employed as covering of the waveguide is positive and higher in absolute value than its own imaginary part and the real part of the permittivity of the waveguide [113]. 

LMRs are not a technology as mature as SPRs [95], but their high sensitivity compared to SPRs [122,123] has already led to the development of biosensors that enable miRNA detection such as hsa-miR-34b-5p (UAGGCAGUGUCAUUAGCUGAUUG, also known as miR-34b) [124] and has-miR-223-3p (UGUCAGUUUGUCAAAUACCCCA, also known as miR-223) [125], although further research is required. Both of them are linked with RA, miR-34b is overexpressed in RA T cells [34]; while increased serum miR-223 levels are considered to be connected with higher disease activity and disease relapse [33]. These miRNAs are not included in Table 1 as both the LOD and the dynamic range have not been studied in depth for the corresponding biosensors.

Regarding silicon photonic microring resonators, a ring resonator is an optical waveguide which is looped back on itself, such that a resonance occurs when the optical path length of the resonator is exactly a whole number of wavelengths [126,127]. The employment of silicon, due to the high refractive index contract between this material and its oxide (or air), enables the development of compact microring resonators [128]. This feature is interesting in biosensing applications, as it allows having several rings on a single chip for multiplexing purposes.

The main feature of the sensors based on silicon photonic microring resonators for the detection of miRNA is not the LOD (which tends to be in the range of nM or pM in the best case) but the fact that they are used in arrays, which permit several miRNAs to be detected at the same time, with different sets of rings or chips functionalized to detect different miRNAs. This fact is especially interesting considering that it has been stated that a sensor to accurately detect rheumatoid arthritis must be based on several biomarkers. 

**Figure 10:**
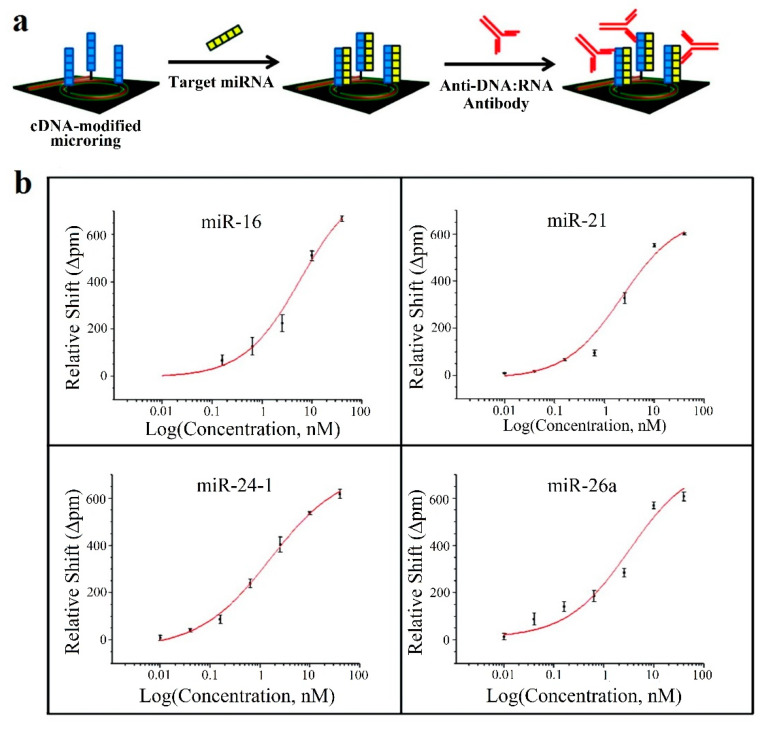
(**a**) Microring resonators with amplification strategy based on using anti DNA:RNA antibodies. (**b**) Calibration curves for miR-16, miR-21, miR-24 (designed as miR-24-1) and miR-26a. Plots were constructed from the relative shifts at 40 min. (**a**,**b**) Adapted with permission [67]. Copyright 2011 American Chemical Society.

This strategy is employed in [65], where 4 miRNAs, all of them linked to rheumatoid arthritis (let-7c-5p, miR-21, miR-24, miR-133b) are detected; in [66], with 7 miRNAs, 6 of which (miR-21, miR-26a, miR-29a, miR-106a, miR-222, miR-335) are related to rheumatoid arthritis; and in [67], where 4 miRNAs (miR-16, miR-21, miR-24, miR-26a), all linked to rheumatoid arthritis in this case. Amplification techniques are also used: enzymatic signal amplification in [66] and amplification using an anti DNA:RNA antibodies in [67] (Figure 10a).

In all the cases [65,66,67], cross-specificity among the miRNAs of the corresponding work is studied, showing that only the rings that have been functionalized with the corresponding complementary probe exhibit a response. A particular specificity assay is presented in [65], where two sets of microrings are functionalized with DNA probes that are complementary to let-7b and let-7c-5p, respectively (these miRNAs only differ in one base). In phosphate-buffered saline (PBS), the specificity assay does not work (both sets of microrings detect both miRNAs), but in a 50% (*v*/*v*) solution of formamide, the assay works (each miRNA only binds to the corresponding set). On the other hand, among these articles, the lowest concentrations are detected in [67], with a value of 10 pM for all miRNAs except for miR-16 (160 pM). The calibration curves of this sensor can be observed in Figure 10b.

Since the association with RA of some of the miRNAs detected using silicon photonic microring resonators has not been given before, it is described below. Regarding let-7c-5p, this miRNA is commonly used in specificity assays of let-7a, for example, in [57,58]; and has been studied in [65]. In [76], the relationships among XIXT (X-inactive specific transcript, a non-coding RNA on the X chromosome), STAT3 and let-7c-5p in RA are analyzed. Let-7c-5p is downregulated in RA cartilage tissues and it is concluded that the overexpression of let-7c-5p may contribute to prevent RA progression, although further studies are required.

In the case of miR-24, levels are reported to be higher in plasma from RA patients compared to healthy controls and also higher than in osteoarthritis (OA) and SLE [35]. Regarding miR-26a, it is overexpressed in PBMCs and plasma of RA patients. However, it is also dysregulated in other diseases, that is, miR-26a is not a specific biomarker for RA [35,74]. With respect to miR-29a, in [80] it is said that miR-29a is downregulated in serum, synovial tissues and fibroblast-like synoviocytes (FLS) of RA patients. This work showed that miR-29a inhibits proliferation and induces apoptosis in RA FLS by targeting STAT3.

Concerning miR-106a, in a case-control study of 21 RA patients and 22 age- and sex-matched healthy controls performed on PBMCs, it was found that miR-106a, among other miRNAs, was downregulated in PBMCs of RA females versus control females [81]. The association of miR-133b with RA is detailed in [35], where it is detected with a 4.34-fold difference in RA patients compared to healthy controls, although it did not fulfil all the requirements to be considered a relevant RA plasma biomarker. Finally, miR-222 expression in PBMCs is significantly elevated in RA patients compared with healthy controls [85], a statement which is also true for miR-335 [86].

### 2.3. Other Optical Sensing Techniques: Interferometry and Surface-Enhanced Raman Spectroscopy (SERS)

Apart from fluorescence-based and resonance-based sensors, interferometry-based sensors have been also presented in literature for miRNA detection. 

Waveguide interferometric sensors are based on the light travelling through 2 different paths. In one of the paths, the sample is placed, changing the refractive index and affecting the evanescent field of the guided mode, thereby inducing a phase shift. The other path is known as the reference arm, where the light propagates without suffering any alteration. The interference of the modes that travel through both paths produces a signal that can be measured at the sensor output, which is related to the analyte concentration in the case of biosensing applications [129].

Their main advantages include their high sensitivity, broad dynamic range and long interaction length, whereas their high sensitivity to wavelength instability, mechanical vibrations and temperature changes are their main inconveniences, therefore requiring coherent and stabilized light sources as well as an isolated environment for being functional [129].

In [68] is described a Mach–Zehnder interferometer which enables the rapid detection (only 15 min) of miR-21 and let-7a through the measurement of the light phase change. It is a label-free sensor and for both miRNAs the LOD is 1 nM, with a linear range from 1 nM to 1 μM [68]. A different case is demonstrated in [69] for the detection of let-7a by means of the utilization of an optofluidic sensor manufactured by aligning a microfiber in lateral contact with a capillary to form a modal interferometer. In this case the LOD achieved is 212 pM with a linear range from 2 nM to 20 μM [69]. 

SERS is a surface spectroscopy technique that consists in combining Raman spectroscopy, which is based in measuring the scattering of light after interacting with the chemical bonds of molecules, and signal enhancement that is provided by the plasmon resonances in the metal substrate [97,120]. 

The peaks in a Raman spectrum are between 10 and 100 times narrower than the emission peaks in fluorescence sensors, making Raman spectroscopy ideal for multiplexing, and therefore able to detect several different biomarkers at the same time [130].

In [70], Ag nanorod array substrates prepared by oblique angle vapor deposition are employed as SERS platform. Here, a partial least square (PLS) regression model is built using SERS for identifying the concentration of different miRNAs (ranges from 6 μM to 150 μM for each) in mixtures that contain several of them: let-7a, miR-16, miR-133a-3p; all linked with RA. The model also enables let-7a concentration to be recovered in assay where miR-16, miR-12, miR-24 and miR-133a are employed as background interferences.

The association of let-7a and miR-16 with RA has been previously explained. Regarding miR-133a-3p, it is a negative regulator of Runx2 (Runt-related transcription factor 2) [82]. In addition to this, miR-133a is also reported to be upregulated in synovial fibroblasts [21,32,34] (although in these references, it is not clear whether ‘miR-133a’ refers to miR-133a-3p or miR-133a-5p). For other miRNAs, these controversies have been solved either because the RNA sequence could be consulted in the corresponding reference and contrasted with [36] or because, in case the RNA sequence cannot be checked, the used name (in this case ‘miR-133a’) is considered a valid name in [36] and can unmistakably be associated with a RNA sequence (which does not happen in the case of ‘miR-133a’).

Gold and silver (Au–Ag) nanoprobes, named nanomushrooms in this case, are employed as SERS probes in [71]. In this work, fragments of DNA of several viruses (hepatitis A, hepatitis B and human immunodeficiency viruses) are detected. Regarding miRNAs, miR-21 detection is individually studied, with a dynamic range from 10 fM to 100 pM. The LOD of this sensor for miRNA detection has been determined to be lower than 10 fM. Then, the simultaneous detection of 3 miRNAs (miR-21, miR-31 and miR-141, all of them linked with RA) is assessed, with their concentrations ranging from 1 pM to 10 nM). These results have been achieved thanks to the use of a different dye (ROX, 4-aminothiophenol, Cy3) with each miRNA. It is also worth mentioning that almost identical results are obtained for miR-21 in both buffer and diluted (20%) human serum, working with a concentration of 100 pM.

**Figure 11:**
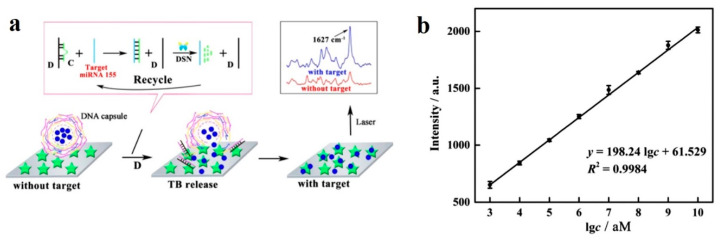
(**a**) SERS sensing of miR-155 using DNA microcapsules and DSN amplification. (**b**) Linear curve of Raman intensity (1627 cm^−1^) for concentrations of miR-155 from 1 fM to 10 nM. (**a**,**b**) Adapted with permission [72]. Copyright 2018 American Chemical Society.

Finally, in [72], SERS is combined with duplex-specific nuclease (DSN) amplification technique, already reported in [38], for the detection of miR-155. The sensor in this work is based on the design of DNA microcapsules that contain toluidine blue (TB), a Raman dye. The presence of miR-155 induces the destruction of the microcapsules, releasing the TB and enabling the detection, which is improved thanks to the DSN amplification (Figure 11a). This biosensor achieves a LOD of 0.67 fM, with a linear dynamic range of 7 decades (1 fM to 10 nM) (Figure 11b), which can be only considered below [47] in terms of dynamic range (this sensor covers slightly more than 7 decades, although it does not follow a linear relationship).

## 3. CRP (C-Reactive Protein)

C-reactive protein (CRP) was first identified in 1930 [131] and it was subsequently considered to be an “acute phase protein,” an early biomarker of infectious or inflammatory conditions. CRP is a protein that is synthesized by hepatocytes and whose production is stimulated by cytokines, particularly interleukin-1 (IL-1), interleukin-6 (IL-6) [14], and interleukin-17 (IL-17) [132], in response to infection or tissue inflammation. Levels in healthy individuals are normally below 10 mg/L [14] while higher levels are associated with a significantly increased prevalence of inflammatory conditions, such as rheumatoid arthritis [15].

In 1982, a study was carried out where CRP levels were measured in 99 patients with RA. The results confirmed that the serum CRP concentration closely reflects activity of RA and is of value in its objective assessment [16]. Therefore, as stated in the introduction of this review, CRP is considered to be a relevant biomarker in the monitoring of RA.

However, in research carried out with more than 39,000 healthy women, it was concluded that a single CRP level is not usable to predict an increased risk of RA [133]. In this sense, CRP is part of one of the five combined disease activity measures of RA that were proposed by the ACR in 2019 [5]. In particular, it is suggested its combination with the Disease Activity Score in 28 joints (DAS28-CRP). The use of CRP in combination with swollen joint count as well as other biomarkers, such as rheumatoid factor (RF) and ACPA (see Section 4); has also been previously proposed for RA diagnosis [134].

The information about the optical biosensors for the CRP detection is summarized in Table 4. In order to homogenize the results and enable their comparison, all the concentrations have been expressed in g/L, regardless of the units employed in each respective work. For more detailed information an additional column (‘Matrix’) has been included here to indicate the medium in which the detection is performed (buffer, plasma, serum). When several media are employed it is also specified to which matrix corresponds the data.

Several sensors for the detection of CRP based on surface plasmon resonance have been found in the literature. In [135], an Au-based SPR sensor coated with a biotin layer and a streptavidin layer is used to detect CRP, achieving a LOD of 1 mg/L in phosphate-buffered saline (PBS). This sensor employs a sandwich strategy, utilizing an antiCRP antibody (antiCRP C6) for the entrapment of the CRP and another one (antiCRP C2) for its detection. 

In the same manner, an SPR sensor with a gold surface is proposed in [136]. Here, the gold surface is functionalized with 1-ethyl-3-(3-dimethylaminopropyl)-carbodiimide hydrochloride (EDC)-activated protein A/G, which possesses four Fc-binding sites from protein A and two from protein G for antibody recognition. In this case, the LOD is decreased by 3 orders of magnitude compared with the previous sensor, achieving a value of 1.2 μg/L. The calibration curves were elaborated in HEPES-buffered saline (HBS), diluted human plasma, diluted human serum, and diluted human whole blood and in all the cases, they were quite similar. Specificity was thoroughly studied using several nonspecific control proteins at high concentrations compared to their physiological levels: human serum albumin (HSA), human lipocalin 2 (LCN2), human fetuin A (HFA), interleukin (IL)-1 β, IL-6, IL-8 and tumor necrosis factor (TNF)-α. The results obtained revealed a response 10 times lower than that corresponding to a CRP concentration of 20 μg/L.

**Figure 12:**
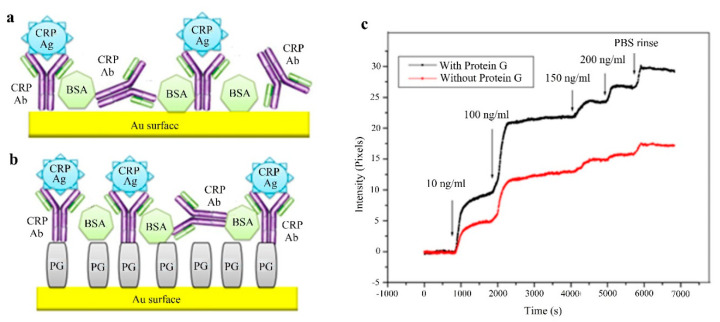
(**a**) Biosensor configuration without protein G. (**b**) Biosensor configuration with protein G. Key: CRP Ab: anti-CRP antibody, CRP Ag: CRP antigen, BSA: bovine serum albumin, PG: protein G. (**c**) SPRi signal versus time for different concentrations of CRP in human plasma: with protein G (black), without protein G (red). (**a**–**c**) Adapted under the terms of the Creative Commons Attribution 4.0 International License (https://creativecommons.org/licenses/by/4.0/) [137]. Copyright 2014, The Authors. Published by Scientific Research Publishing Inc.

A similar approach is shown in [137] employing SPR imaging (SPRi) and protein G. Two methods are used: a simple and fast physical adsorption (Au surface with immobilized anti-CRP antibody without protein G, see Figure 12a), and an oriented immobilization antibody strategy, that differs from the former in using protein G between the Au surface and the anti-CRP antibody, see Figure 12b. In both strategies the bovine serum albumin (BSA) acts as a blocker. The studied range covers from 50 ng/L to 100 μg/L (3 orders of magnitude) without protein G and from 10 ng/L to 100 μg/L (4 orders) with protein G and the respective LODs are 50 ng/L (without protein G) and 10 ng/L (with protein G). The results are better with the second method as the binding capacity of the anti-CRP antibody is directly dependent on the surface density of protein G. Specificity is assessed with rabbit antigen, which produces no relevant response. Previous results correspond to PBS solution. Regarding the experiments performed with the device in diluted human plasma, the LODs obtained are 10 μg/L and 5 μg/L without and with protein G (detection range in Figure 12c), slightly higher than in [136].

There are also sensors based on LSPR, which can be viewed as a particular type of SPR, as previously mentioned. In particular, a label-free LSPR sensor that uses a 96-well plate and measures the optical density (OD) value change is presented in [138]. The sensor has a 3D nanocup array structure covered by a Au-TiO_2_-Au multilayer and it is immersed in a 11-mercaptuoudecanoic acid (MUA) solution to form a self-assembled monolayer. This structure is combined with a sandwich detection strategy that uses two antibodies (as described in [135]) and it achieves a LOD of 50 μg/L in PBS, with a linear range from 50 μg/L–25 mg/L. This sensor was also tested with diluted (10 times) blood serum samples, providing in this case a LOD of 3.1 mg/L.

Similar results (LOD of ~50 μg/L in PBS buffer) were achieved in [139]. In this case, the LSPR sensor utilizes Au NPs grafted with poly(2-methacryloyloxyethyl phosphorylcholine) (PMPC) as an artificial CRP recognition layer. This layer was prepared with atom transfer radical polymerization (ATRP). This biosensor was tested in PBS buffer and 1% diluted human serum, obtaining a very similar dynamic range (50 μg/L–3 mg/L) with both media. Gold nanoparticles are also employed in [140], where the LSPR sensor chip is based on a cuvette cell system with a substrate modified with (3-aminopropyl)triethoxysilane (APTES) and functionalized with cysteine-protein G. The LOD obtained was 11.28 μg/L with a linear range from 10 μg/L to 10 mg/L in PBS. The specificity was assessed with proteins: hemoglobin (Hb), transferrin (TF) and HSA; both separately and in mixture, and in all the cases the response of the LSPR sensor was around 5 times lower than that corresponding to CRP. 

**Figure 13:**
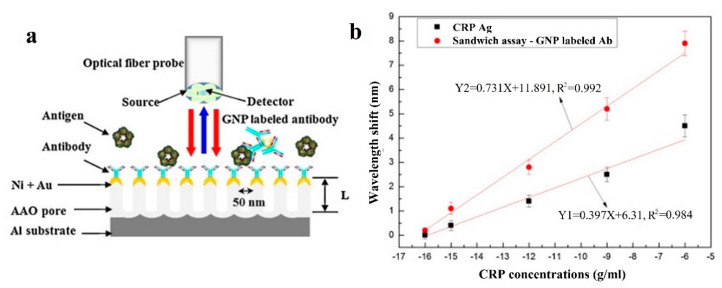
(**a**) Schematic cross-sectional figure showing the structure of a fabricated anodicaluminum oxide (AAO) chip for CRP detection. (**b**) The linear regression of the resonance wavelength shift after CRP antigen–antibody reaction (black squares) and after gold nanoparticle labelled CRP secondary antibody reaction (red circles). (**a**,**b**) Reprinted [141], Copyright 2013, with permission from Elsevier.

Finally, gold nanoparticle-labelled antibodies are used in [141]. In this case, the sensor is based on a nanostructured anodicaluminum oxide (AAO) substrate (Figure 13a). In order to increase the sensitivity, a sandwich assay with a gold nanoparticle-labelled secondary antibody (similar to the strategy used in [135,138]) was undertaken, reducing the LOD from 1 pg/L to 100 fg/L in Tris-HCl buffer. The range is linear over 10 decades (100 fg/L–1mg/L) although there are only 5 points (measured 10 times each) in this range (Figure 13b). This optical sensor is the one with the lowest LOD (100 fg/L) and the largest dynamic range (10 decades) for CRP detection included in this review.

There is still one more sensor based on resonances that enables the detection of CRP and is not a SPR or LSPR sensor but an LMR sensor. As previously mentioned, LMRs are still in their infancy, although their high sensitivity makes them attractive for biosensing [151,152,153], leading to applications such as an immunoglobulin G (IgG) biosensor [154].

In [142], LMR are generated using an indium tin oxide (ITO) thin-film fabricated onto the planar region of D-shaped fiber using the Direct Current (DC) sputtering technique. An additional aptamer-based sensitive and selective layer is fabricated onto the ITO coating using the layer by layer technique. This device achieved a LOD of 62.5 μg/L in Tris-buffered saline (TBS) buffer and high specificity when compared with urea and creatinine (irrelevant response of the sensor in these cases). LMR have also been employed in [155] to develop a label-free D-dimer biosensor, another biomarker which, as CRP, is correlated with inflammation and RA [156].

Another sensor based on the change of the refractive index is introduced in [143]. It is a label-free metal clad leaky waveguide (MCLW) sensor covered with a nitrocellulose membrane deposited via spin coating. The lowest concentration detected in human serum with this sensor is 100 μg/L.

On the other hand, several CRP sensors based on etched Fiber Bragg gratings (eFBG) can be found in the literature. Fiber Bragg gratings (FBG) consist in periodic perturbations of the refractive index along the fiber length formed by exposure of the core to an intense optical interference pattern [157]. FBGs enable strain and temperature to be measured in many applications but are insensitive to the changes of the refractive index of the surrounding medium as light coupling only happens between core modes, which are shielded from the influence of the medium thanks to the fiber cladding. In order to modify this condition and permit the utilization of FBGs for biosensing purposes, it is necessary to perform an additional post-processing step. In this sense, a typical approach consists in reducing the cladding thickness around the grating region via an etching process, resulting in an eFBG, also known as thinned or reduced cladding FBG [158]. Once the cladding has been removed, the evanescent field goes beyond the fiber, therefore the resonance wavelength is affected by the surrounding refractive index, enabling a correlation between the analyte concentration and the sensor output to be established. The smaller the diameter of the fiber, the higher the sensitivity, although the resulting structure is more fragile [159]. 

In [144], an eFBG coated with graphene oxide (GO) and anti-CRP antibody enables to detect CRP with a LOD of 10 µg/L by monitoring the shift in the Bragg wavelength. The employment of GO increases the sensitivity of the sensor by a factor of approximately 5. The sensor possesses a linear range over 4 decades (10 μg/L–100 mg/L) in deionized water and the specificity has been assessed with urea (1.8 g/L), glucose (3.8 g/L) and creatinine (5.8 g/L); which do not appreciably affect the sensor operation.

**Figure 14:**
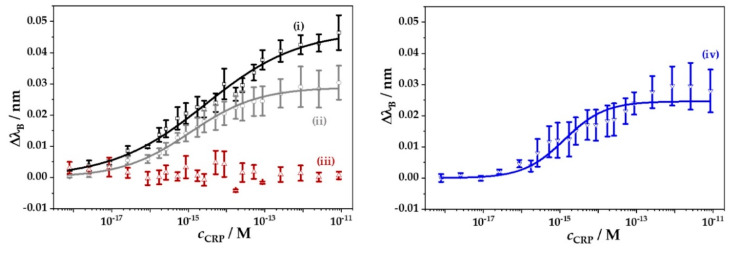
The Bragg wavelength shift ∆λB of biofunctionalized eFBG fibers as a function of CRP concentration (i) without any interfering substances (black), (ii) in the presence of the interfering substances urea (1.8 g/L) and ascorbic acid (1.8 g/L) (grey), (iii) without fiber coupling of the CRP-specific aptamer and any interfering substance (brown), and (iv) in presence of diluted CRP deficient human plasma (blue). Data were fitted to the Langmuir–Freundlich isotherm. Reproduced under the terms of the Creative Commons Attribution 4.0 International License (https://creativecommons.org/licenses/by/4.0/) [145]. Copyright 2018, the Authors. Published by MDPI.

The LOD is reduced until 0.82 pg/L in [145], where the gratings have been fabricated point-by-point via a non-linear absorption process of a highly focused femtosecond pulsed laser and the etching has been done with hydrofluoric acid. This LOD corresponds to a modified aptamer buffer, although in human plasma (diluted CRP deficient human plasma that has been spiked with CRP) the LOD is also quite low, with a value of 27.6 pg/L. The biofunctionalization has been carried out with a single stranded DNA aptamer specific for CRP. The dynamic range of the sensor covers from 0.8 pg/L to 1.2 µg/L in buffer (this range corresponds with a 7 aM–10 pM range). The biosensor shows a high specificity to CRP even in the presence of interfering substances (urea and ascorbic acid) and has also been tested with diluted human plasma (Figure 14).

In [146], a sensor that uses reflectometric interference spectroscopy (RIfS) is reported. RIfS is based on white light interference. Changes in the optical thickness of the layer between two interfaces, due to the swelling of a polymer or the binding of an analyte, are detected thanks to the variation in the interference pattern [160]. The sensor described in [146] employs 2 layers: an interference layer, that is, a TiO_2_ layer which is placed on a silicone substrate by liquid phase deposition (LPD); and a sensitive layer, that is, another TiO_2_ layer which is immersed in a LPD solution containing poly(L-lysine) (PL) and anti-CRP antibody. The lowest concentration detected here is 100 µg/L in HBS-P buffer (HBS buffer with 0.005% *v/v* surfactant P20).

**Figure 15:**
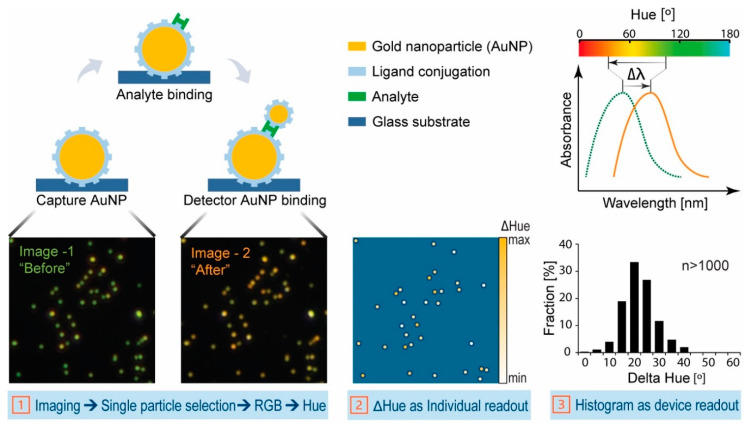
Schematic of the sensing platform using a swarm of single nanoparticle colorimetric sensors. Reprinted [147], Copyright 2019, with permission from Elsevier.

A swarm biosensing platform is utilized for detecting CRP in [147]. Computational image registration and analysis is performed to determine the hue (one of the main properties of a color) change associated to the surface plasmons from thousands of single Au NPs. An image is taken when only the capture Au NPs are immobilized (“before”, Figure 15—1 Image-1) and when the binding of analyte (CRP) and detector Au NPs has taken place (“after”, Figure 15—1 Image-2). These images enable the delta hue of each individual nanoparticle to be calculated (Figure 15—2). The results for all the particles are plotted in a histogram representing the sensor readout, that can be associated with the CRP concentration (Figure 15—3). The LOD achieved in deionized water is 1 μg/L with a linear range of 4 decades (from 1 μg/L to 10 mg/L) in deionized water. The sensor operation has also been tested in human serum spiked with CRP achieving a proper detection in concentrations of 1 mg/L, 3 mg/L and 10 mg/L.

Au NPs are also used in [148], although in this case the sensor is based on photoluminescence. It consists in an optical “turn off” nanosensor (also known as molecular beacon), made of a DNA aptamer with a quantum dot (QD) attached on the 5′ terminal and a gold nanoparticle attached on the 3′ terminal and based on fluorescence resonance energy transfer (FRET). The rise in the CRP concentration decreases the photoluminescence intensity resulting in an increase in quenching efficiency. This device achieves a LOD of 45 ng/L, with a linear range of 75 ng/L–1.65 mg/L in 100 times diluted PBS, and it was also tested with CRP spiked human serum, achieving a detection in the order of 100 ng/L. The specificity has been assessed with control proteins: glycated albumin (GA), thrombin, TF and TNF-α; all of which show negative quenching (the other way round from CRP) except for TNF- α, with a 10 times lower positive quenching when compared to CRP; therefore, the sensor is highly specific.

QDs are also used in [149]. This sensor is based on a lateral flow immunoassay (LFIA) in which a double antibody sandwich technique (also employed in [135,138,141]) and fluorescent cadmium telluride (CdTe) QDs are employed. In particular, LFIA is developed on a nitrocellulose strip, Staphylococcal protein A (SPA) is used for the biofunctionalization and anti-rabbit IgGs are utilized as control. Measurements were mainly carried out in Tris buffer (13 measurements), although some more (5 measurements) were taken in human serum spiked with CRP (all in the range 1–300 mg/L). The achieved LOD (0.3 mg/L in buffer) is not far below the clinical cut-off value of CRP concentration for RA (10 mg/L). However, the main interest of this work is that two RA biomarkers are detected, not only CRP, but also IL-6. This biomarker will be treated in the following section.

**Figure 16:**
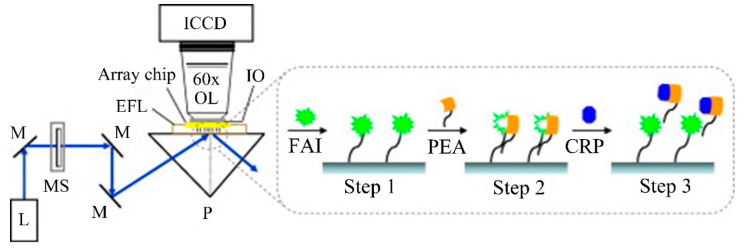
Laboratory-built TIRFM system used for the MSF-based detection of CRP. Key: L, laser; M, mirror; MS, mechanical shutter; P, prism; IO, immersion; OL, objective lens; EFL, evanescence field layer, ICCD, intensified charge-coupled device. Reprinted [150], Copyright 2010, with permission from Elsevier.

Finally, another sensor that involves fluorescence is explained in [150]. Its mechanism relies on molecular switching fluorescence (MSF), studied by means of total internal reflection fluorescence microscopy (TIRFM), already mentioned in [42]. The fluorescence of fluoreseinamine isomer 1 (FAI), which is printed on a 3-glycidoxypropyl-trimethoxysilane (GPTS)-coated glass coverslip (Figure 16 Step 1), is decreased upon binding with GPTS-conjugated O-phosphorylethanolamine (PEA) (Figure 16 Step 2). Then, it increases linearly with CRP concentration (Figure 16 Step 3). The LOD achieved is 20 pg/L, with a linear range from 20 pg/L to 12.5 ng/L in PBS buffer. The specificity is studied here using albumin (2 pM), producing no appreciable change in the sensor response. Some tests were undertaken with human serum samples with CRP concentrations in the 625 ng/L–1.25 μg/L range, achieving good results.

## 4. Other Biomarkers

In this section, a few more RA key biomarkers for which optical sensors have been found are described. Although in these cases there are not as many optical sensors as for miRNA and C-reactive protein, the rheumatoid factor (RF) and the ACPA are widely mentioned in rheumatoid arthritis literature [161,162,163] while histidine and IL-6 are the other two RA biomarkers covered in this section. The information about the optical biosensors explained in this section is summarized in Table 5.

Furthermore, research continues being carried out in order to find new biomarkers for rheumatoid arthritis, including proteins as 14-3-3 η [164], enzymes as secretory phospholipase A2 group IIA (sPLA2-IIA) [165], or polymorphisms as rs688136 from the vasoactive intestinal peptide (*VIP*) gene [166], or rs13192471 from the *HLA-DRB1* gene [167].

### 4.1. Rheumatoid Factor (RF)

RFs are a family of autoantibodies directed to the Fc portion of immunoglobulin IgG [161]. They are locally produced in RA by B cells present in lymphoid follicles and germinal center-like structures produced in inflamed synovium. IgM RFs are the most frequently detected isotype (they are detected in 60–80% of RA patients) but IgG, IgA, IgE, and IgD RFs can also be observed. 

RFs can be detected in patients with many non-rheumatic conditions or even in the healthy population, and they are frequently detected in patients with systemic autoimmune diseases, such as SLE or mixed connected tissue disease. Nevertheless, RF testing in RA patients has a sensitivity (how many sick patients are identified as such) between 60% and 90% and a specificity (how many healthy patients are identified as such) of 85% [25]. Furthermore, the detection of the different RF isotypes can also help with the diagnosis. In particular, it has been observed that an increase in both IgM and IgA RFs possesses high specificity (99%) and at the same time low sensitivity (47%) for RA [173].

The ACR (formerly, the American Rheumatism Association) had already established RF testing as one of the classification criteria for RA in 1987 [174]. Nowadays, it continues being considered as a relevant factor for RA assessment for both the ACR and the European League Against Rheumatism (EULAR) [175]. 

At the present time, the clinical threshold of RF for RA diagnosis is admitted to be around 20 IU/mL [176]. An international unit (IU) is the amount of a substance (vitamin, hormone, enzyme, medicine …) that has a certain biological effect, and this unit is employed to measure its activity. For each substance there is an international agreement on the biological effect that is expected for 1 IU [177]. In 1970, the National Institute for Medical Research (London, England), as a result of a request by the World Health Organization (WHO) Expert Committee on Biological Standardization, established the international reference preparation of RA serum and defined the IU for RA serum as the activity contained in 0.171 mg of the international reference preparation [178]. Despite mentioning ‘RA serum’, this IU definition is used for RF and the WHO employs this procedure to calibrate assays and diagnostic test kits which measure RF levels in patient serum. Nevertheless, the WHO Expert Committee on Biological Standardization has endorsed a proposal to develop a second WHO International Standard for RF [179].

**Figure 17:**
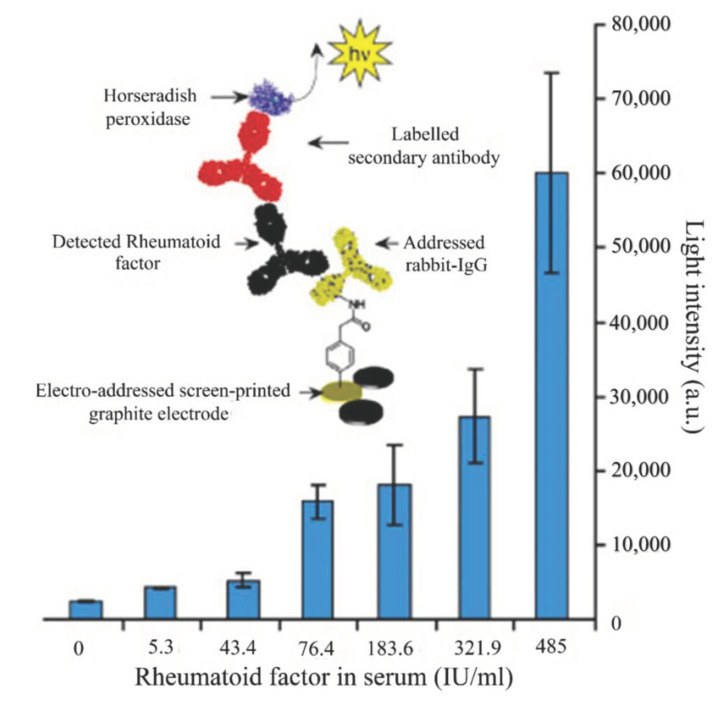
Schematic representation of the capture immunoassay performed on a screen-printed graphite electrode. Histogram: chemiluminescent detection of RF in human sera using screen-printed (SP) microarrays. Reprinted [168], Copyright 2007, with permission from Elsevier.

In [168], a sensor based on chemiluminescence is presented for the detection of rheumatoid factor. Rabbit immunoglobulins G (IgG) are modified with a diazotated aniline derivative (diazotated CMA) and electro-grafted to a carbon paste screen-printed (SP) microarray composed of eight working electrodes. The immobilized rabbit IgGs are used to capture RFs, which are subsequently linked to peroxidase-labelled antibodies in order to produce a measurable signal (see Figure 17), that is, a sandwich detection technique, already seen in the case of CRP [135,138,141]. This sensor enables RF to be detected in the range from 5.3 to 485 IU/mL from samples of human serum diluted 10 times in modified PBST (PBS with 0.1% surfactant Tween 20, including an additional 1% BSA) buffer, see Figure 17. Here, it is important to remark that the lowest RF concentration detected (5.3 IU/mL) is below the clinical threshold (20 IU/mL). 

### 4.2. Anti-Citrullinated Protein Antibodies (ACPA)

ACPA are a group of autoantibodies directed against citrullinated proteins/peptides. ACPA include anti-keratin antibodies (AKAs), anti-perinuclear factors (APFs) or anti-Sa antibodies [161]. Before explaining more concepts related to ACPA, the term ‘anti-cyclic citrullinated peptides’ (anti-CCP) has to be clarified. ACPA are usually tested with anti-CCP assays [169,170,175] and these two concepts (ACPA, anti-CCP) are usually treated as synonyms, without explaining the difference between both. In fact, it seems the term ‘ACPA’ appeared to substitute the term ‘anti-CCP’, as new assays also detect non-cyclic citrullinated peptides [180]. However, the term ‘anti-CCP’ continues being used, which can lead to confusion.

ACPA were established as a criteria for rheumatoid arthritis assessment by the American College of Rheumatology (ACR) and EULAR in 2010 [175]. ACPA have a high specificity (90–95%) in RA diagnosis and they are considered superior to RF in this aspect [161,181], while their sensitivity is between 60% and 75% [181]. These values are a general reference for ACPA and may vary when considering a particular type of antibody among ACPA. 

On the other hand, there are several assays for measuring ACPA, and the thresholds for positive results vary, so it is difficult to provide a cut-off value [180]. In [175], the following definitions are given for an ACPA test (also valid for RF tests) that can be applied to solve the previous problem: negative, if the IU values are less than or equal to the upper limit of normal (ULN, a threshold or cut-off value) for the laboratory and assay; low-positive, if the IU values are higher than the ULN but ≤ 3 times the ULN for the laboratory and assay; high-positive, if the IU values are >3 times the ULN for the laboratory and assay.

However, there is controversy about the prognosis value (related to the progression of the disease) of ACPA, not to be mistaken with their diagnosis value. It has been stated that RF, and not ACPA, is associated with disease activity in RA. Disease activity is a measure of the progression of RA and considers the number of swollen joints or the levels of acute-phase reactants [182].

**Figure 18:**
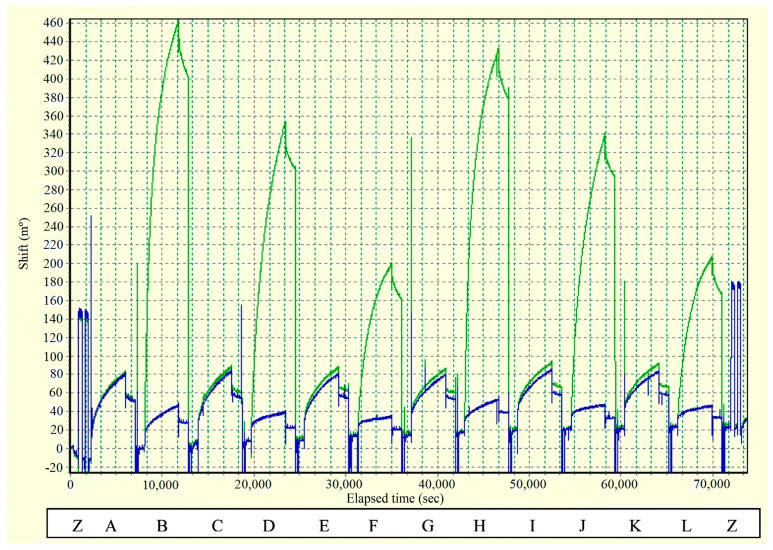
Sensorgram of citrulline peptide containing spot (green) and its arginine control spot (blue). The array was probed two times with three different RA sera: (**B**,**H**) serum 1, (**D**,**J**) serum 2, and (**F**,**L**) serum 3. Between probes, normal sheep serum was used (**A**,**C**,**E**,**G**,**I**,**K**) to increase the number of regenerations. After every serum (RA or sheep serum) the sensor was regenerated with 10 mM glycine‚ HCl. Reprinted with permission [169]. Copyright 2007 American Chemical Society.

Several works have been found in the literature where ACPA are detected utilizing SPRi. In [169], a microarray imaging system, which measures the SPR dip angle, is employed to determine the presence of ACPA in the sera of 50 RA patient. Sera from 29 more patients, including patients with other related diseases and healthy individuals, is used as control. Here, a 24 spot microarray with a *N*-hydroxysuccinimide (NHS) preactivated polycarboxylate-coated gold sensor surface is used. It contains two types of citrullinated peptides, two other control peptides (containing arginine instead of citrulline) and human IgG. After the incubation of the serum, which is diluted 50 times in PBS, only the citrullinated peptides show a measurable response, due to their binding with ACPA (see Figure 18). The lowest ACPA concentration detected with this setup is 0.5 pM.

On the other hand, in [170], 20 citrullinated peptides (as well as the corresponding non-citrullinated control peptides) were immobilized on a 48-spot microarray. Binding of ACPA with the citrullinated peptides was studied with SPRi, employing sera (1:50 dilution in PBS) from 374 early RA patients. As expected, sera from patients with ACPA generally showed more reactivity with citrullinated peptides than with the corresponding control peptides, that is, the angle shift was between 3 and 6 times higher in the case of the citrullinated peptides compared to control peptides in more than 50% of the cases. This work also confirmed the heterogeneity of the ACPA response in RA and revealed 12 distinct ACPA profiles. However, associations between ACPA profiling in early RA and disease activity or progression scores were not found, which is coherent with [182], that is, ACPA are not associated with baseline disease activity in RA.

### 4.3. Interleukin-6 (IL-6)

IL-6 is a pleiotropic cytokine that is deregulated in many inflammatory and autoimmune diseases, including RA. In particular, IL-6 plays a crucial role in RA pathophysiology. IL-6 is found in abundance in the synovial fluid and serum of patients with RA and its level correlates with the disease activity and joint destruction [183]. As a reference value, the level of serum IL-6 is typically less than 10 pg/mL in healthy adults [184].

Due to its multiple effects, IL-6 is involved in the various phases of RA development, including the acute phase, the immuno-inflammatory phase, and the destructive phase. IL-6 may also be mediating many of the systematic manifestations of RA, including CRP. Moreover, IL-6 may contribute to the induction and maintenance of the autoimmune process through B-cell proliferation, which causes the production of RFs and ACPA as well as Th17 differentiation [185].

Therefore, IL-6 blockade is regarded as a desirable therapeutic option in the treatment of RA. Different IL-6 inhibitors have been studied, such as sarilumab, ALX-0061 or sirukumab; although right now tocilizumab (TCZ) is considered the most relevant one [186].

Despite its connection with RA, the use of IL-6 on its own as a biomarker to diagnose this disease is considered unlikely. Although cytokines, such as IL-6, have clinical utility in other diseases, further characterization may need to be done before employing them as RA biomarkers. Right now, the existing limitations include the RF interference in cytokine assays and understanding the effect that one cytokine may have on the other [187].

**Figure 19:**
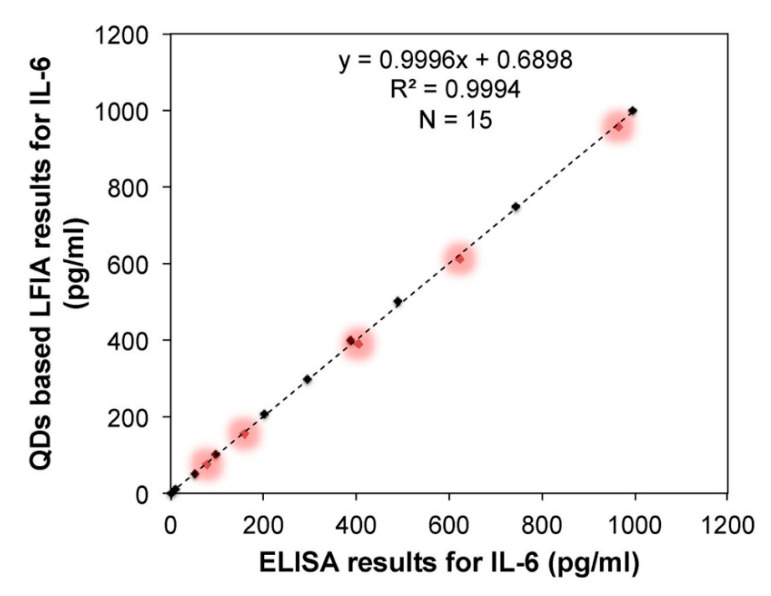
Comparison of lateral flow immunoassay (LFIA) results with standard enzyme-linked immunosorbent assay (ELISA) results. Black points correspond to IL-6 detection in Tris buffer while points highlighted in red correspond to IL-6 spiked in human serum. Reprinted [149], 2019), Copyright 2019, with permission from Elsevier.

IL-6 detection is performed with the biosensor described in [149], a biosensor that has already been mentioned as it also enables CRP detection. Here, it is important to remark that the antibodies used for CRP and IL-6 detection and capture are different. As already explained in Section 3, this device utilizes a LFIA on a nitrocellulose strip functionalized with SPA and combined with CdTe QDs and the double antibody sandwich strategy. In the case of IL-6, the LOD obtained is 0.9 pg/mL (below the reference value of 10 pg/mL) with a linear range from 1 pg/mL to 1 ng/mL (3 decades) in Tris buffer (observe the black colored points in Figure 19). Additionally, 5 unknown concentrations of IL-6 spiked in human serum were also estimated (red colored points in Figure 19).

### 4.4. Histidine

Histidine is a semi-essential amino acid, which means the body normally produces as much as it needs. This amino acid is important for the maintenance of myelin sheaths that protect nerve cells and it is metabolized into the neurotransmitter histamine. Histidine is also involved in blood cell manufacture and tissue protection against damage caused by radiation and heavy metals [188].

Early studies of rheumatoid arthritis (1950–1980) reported decreased histidine levels in sera and plasma from RA patients compared with healthy individuals and patients with acute and chronic illnesses other than RA [189,190,191]. On the other hand, it was also stated that low histidine levels were acquired with the disease [192]. Therefore, histidine levels cannot be used as a biomarker to predict the development of the disease on healthy patients, but they enable distinguishing between patients with RA and patients with other diseases or healthy individuals. Based on [190,191,192], a value of around 14 mg/L (approximately 90 μM) of histidine in serum could be used as a reference, with lower concentrations corresponding to RA patients. However, it must be considered that, in these studies, histidine levels are always in a narrow range (12–19 mg/L) with only slight differences between RA patients and healthy individuals.

There also recent studies that suggest the use of histidine as RA biomarker. In [193], the purpose was to identify metabolites (products and intermediates of cellular metabolism) associated with disease activity in plasma and urine from RA patients. Histidine and guanidoacetic acid in plasma and hypotaurine from urine were determined to be the most relevant ones using a multiple logistic regression (MLR) model. Similarly, in [194] metabolite profiling (metabolomics) is proposed as a potentially useful technique for diagnosing RA. Here, in a validation study with 14 RA patients and 20 healthy controls, a set of 52 blood metabolites achieves a sensitivity of 93% and a specificity of 70% RA and it is also considered that a decreased level of histidine is one of the most specific metabolic markers for RA in the study.

Furthermore, a recent work establishes a correlation between low levels of histidine-rich glycoprotein (HRG), a glycoprotein with a high concentration of histidine; and high levels of RF, which suggests the use of HRG as a biomarker for rheumatoid arthritis [195].

Concerning the detection of histidine, in [171] a fluorescent sensor has been developed based on copper(I)-catalyzed azides and alkynes cycloaddition (CuAAC, a type of click reaction). In the absence of histidine, copper(II)-induced ascorbate oxidation takes place and triggers the CuAAC reaction between the weak fluorescent 3-azidocoumarin and propargyl alcohol, forming a strongly fluorescent compound (1,2,3-triazole). The presence of histidine inhibits the first step of this process, causing a drop in the fluorescence. Therefore, the higher the concentration of histidine, the lower the fluorescence. This sensor possesses a LOD of 76 nM in PBS buffer, with a linear range from 500 nM to 100 μM. The specificity was assessed with several natural amino acids in a concentration of 200 μM (lysine, threonine, methionine, valine, tryptophan, tyrosine, histidinol, alanine, phenylalanine) and some common metal ions in concentrations of 100 μM and 1 mM (Zn, Ca, Cd, Pb, Co, Ni, Fe, Al) and in almost all cases the relative fluorescence change was at least 5 times lower than that for a 75 μM histidine concentration.

**Figure 20:**
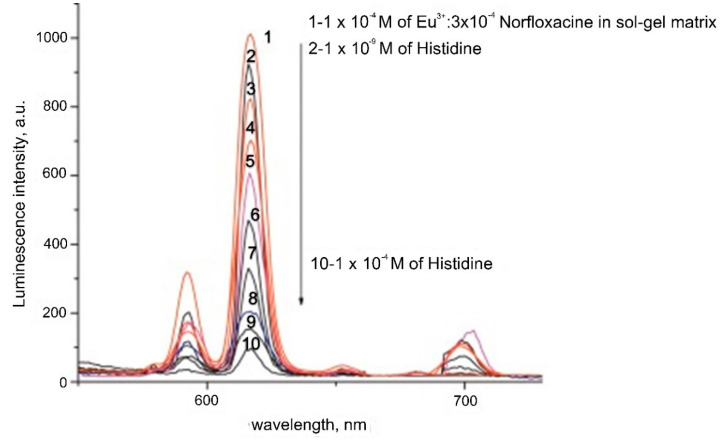
Luminescence emission spectra of nano Eu–norfloxacine complex doped in sol–gel matrix in the presence of different concentrations of histidine in acetonitrile (curve 1: control, curve 2: 1 nM–curve 10: 100 μM). Reprinted [172], Copyright 2015, with permission from Elsevier.

A nano optical sensor, based on the quenching of the luminescence intensity of a Eu–norfloxacine complex doped in sol gel matrix, is presented in [172]. In this case, the intensity at the peak wavelength (617 nm) also decreases with the concentration of histidine. The LOD achieved is 0.6 nM, with a dynamic range from 1 nM to 100 μM in human plasma (diluted with citrate solution, PBS and acetonitrile), see Figure 20. This range is linear in the 1 nM–5 μM interval. The sensor operation was assessed with human serum samples from healthy individuals; Histidinemia, Alzheimer and RA patients; and inflammation in obese women (diseases in which histidine levels are deregulated); with recovery rates between 82% and 118.1%. The best results are achieved for Histidinemia (characterized by high histidine levels), with recovery rates between 99.37% and 101.0%. In the case of RA, the recovery rates range from 90.00% to 118.1% (they correspond to 10 human samples in the 9–28 nM range).

## 5. Conclusions and Outlook

Different biomarkers (miRNAs, CRP, rheumatoid factor, ACPA) associated with rheumatoid arthritis have been described, paying attention to the optical-sensing techniques (fluorescence, absorbance, different types of resonances, interferometry or spectroscopy) proposed in the literature for its detection as well as providing a justification for their use as RA biomarkers. These relationships are not trivial, as some of the examined biomarkers are also related to other diseases and their particular connection with RA is obviated. Therefore, the corresponding optical sensors usually go unnoticed when specifically searching for RA.

The number of developed sensors, as well as the research for new biomarkers, confirms the increasing relevance of biomarkers in the diagnosis of diseases. The employment of biomarkers adds objectivity to the evaluation of the medical state, removing or at least reducing dependence on the doctor or the patient’s assessment, which are more subjective and are, however, still quite commonly used in the diagnosis of rheumatoid arthritis.

However, there are still several challenges that need to be addressed. In the first place, in RA, relying on a single biomarker for diagnosis cannot be considered an adequate procedure. For almost every biomarker, whatever the agreement of its relevance among the scientific community, a work can be found where its importance is put into question due to the obtained experimental results.

Therefore, several biomarkers need to be employed at the same time to provide a precise evaluation. Some efforts have already been made in this direction, especially in the medical field, identifying sets of biomarkers. Nevertheless, there is still work to do, developing more sensing platforms that enable several biomarkers to be detected simultaneously, as has been shown in several works mentioned in this review.

On the other hand, biosensors for miRNA detection, considered the most promising biomarkers, have been tested with tumor cell lines with good results. These assays are performed because the miRNA association with cancer tends to be emphasized, but their relationship with rheumatoid arthritis is usually forgotten. In this sense, very few miRNA optical biosensors have been tested with clinical samples (plasma, serum), an important step towards their use for RA diagnosis. However, as has been demonstrated with other more consolidated biomarkers, such as CRP, the detection of miRNAs for RA diagnosis is only a matter of time.

Finally, it is important to remark that optical biosensors based on different technologies have proven capable of detecting biomarkers linked with RA with decreasingly low LODs, dynamic ranges of several decades, and high specificity, as has been revealed in this review. As a result, they are becoming a key technology that enables a reliable, highly sensitive, reusable, and fully automated solution to be provided for the diagnosis of RA through the simultaneous detection of multiple relevant biomarkers.

## Figures and Tables

**Table 1:** Optical biosensors for the detection of microRNAs (miRNAs) linked with rheumatoid arthritis (RA).

Optical Technique/ Principle	Target miRNA Linked with RA	Linear Dynamic Range	LOD	Specificity Assays	Comments	Ref.
Fluorescence	miR-21	10 nM–10 µM	10 nM	One base mismatched miR-21 and other non-related sequence	Sensor based on PVDF paper impregnated with PT as luminescent reporter	[37]
		50 pM–1 nM (fluorescence)	50 pM (fluores-cence)	let-7e, let-7i, miR-141, single-base-mismatched miR-21	Dual mode sensor (fluorescence and colorimetry) based on FAM labelled hairpin probes, Au NPs and DSN signal amplification	[38]
		125 pM–1.5 nM	125 pM ^(b)^	Blank, let-7a, let-7b, let-7c-5p, miR-21 complementary seq.	Detection strategy that uses CHA, graphene oxide (GO) and hairpin probes with FAM	[39]
		0.2–20 nM	98 pM	miR-26a, miR-122, miR-141*	Method based on rGO, Eosin Y and magnetic silicon microspheres.	[40]
		1–16 nM	47 pM	Mismatched miRNAs (1, 2, 3 or 5 bases) and miR-126*	Sensor based on fluorescence anisotropy (FA) that uses GO-assisted CHA and TAMRA	[41]
		5–100 pM	5 pM	miR-214	Single molecule detection (SMD) assay based on total internal reflection fluorescence microscopy (TIRFM) that uses YOYO-1	[42]
		2 pM–10 nM	2 pM ^(b)^	let-7a, let-7b, let-7c-5p	Sensor that employs GO nanoplates, RCA, triple-helix probes, and FAM.	[43]
		1 pM–10 nM ^(a)^	1 pM	Blank, one and two-base mismatched miR-21, miR-155	MiRNA detection based on RCA reaction, GO and nicking enzyme amplification	[44]
		1 pM–1 nM	1 pM ^(b)^	Blank, miR-210-3p, miR-214	Switch platform using GO and SYBR Green I based on isothermal enzyme free amplification	[45]
		0.6 pM–1.0 nM ^(a)^	0.6 pM	-	Sensor based on photonic crystal enhanced fluorescence (PCEF) that employs Cy5	[46]
		100 fM–5 µM ^(a)^	35 fM	-	MiRNA detection by CXFluoAmp method with CdSe nanocrystals and Rhod-5N	[47]
		10 fM–10 pM	3 fM	Blank, miR-210-3p, miR-214	Sensor that combines isothermal exponential amplification, GO and SYBR Green I	[48]
		2–200 fM	200 aM	Blank, miR-210-3p, miR-214	QD labelled strip sensor based on target- recycled non-enzymatic amplification	[49]
	let-7a	5–300 nM ^(a)^	3.5 nM	let-7c-5p-5p, let-7e, let-7f (based on Tm)	MiRNA detection using carbon nanoparticles and DNA probes labelled with FAM	[50]
		1 pM–5 nM ^(a)^	1 pM ^(b)^	let-7b, let-7e, let-7f, let-7g, let-7i	Assay based on HCR reaction coupled with GO and DNA probes with FAM	[51]
		60 fM–12 pM	10.8 fM	let-7b, let-7c-5p-5p, miR-21	MiRNA detection based on amplification using GO and SYBR Green I	[52]
		10 fM–2 pM	4.2 fM	let-7b, let-7e, let-7f, let-7g, let-7i	Detection platform that uses GO, helicase amplification of HCR and DNA with Cy3	[53]
	miR-141	1 pM–5 nM	1 pM	Single mismatched miR-141, miR-21, miR-200b, miR-429	Sensor based on a β-Ni(OH)_2_ nanosheet, DSN amplification with FAM and TAMRA	[54]
	miR-21	-	10 nM	-		
	miR-21, miR-155	1 pM–1 nM (both)	1 pM (^b)^ (both)	Blank, miR-210-3p, miR-214	Nano-photon switch based on QD and GO for multiple miRNA detection by FRET	[55]
	miR-21 ^(c)^, miR-16, miR-31, miR-155	1 pM–10 nM ^(a)^ (miR-21)	0.4 pM (miR-21)	Cross specificity among all, miR-16 and two one-base mismatched miR-21 (miR-21)	Fluorometric system using rolling circle amplification (RCA), GO and fluorophores.	[56]
	miR-9 ^(c)^	500 fM–300 pM	500 fM (LOQ)	-	45 miRNAs studied in 16 tissues using a 5-laser single molecule detection platform	[57]
	let-7a	-	1 pM ^(b)^	let-7b, let-7c-5p-5p, let-7d		
	miR-125a ^(c)^	10 fM–100 pM	10.3 fM	One and two-base mismatched miR-125a	Detection based on rGO-assisted rolling circle amplification (RCA) and SYBR Green I	[58]
	let-7a	-	100 fM ^(b)^	let-7b, let-7c-5p, let-7d		
	cDNA miR-126 (miR-126 is fixed)	20 fM–100 pM	∼3.0 fM	cDNA miR-126 with mismatched bases (1, 2 or 3), cDNA let-7d, cDNA miR-21, cDNA miR-122, cDNA miR-141	Method using GO, DNA probe with FAM and site specific cleavage using RsaI endonuclease	[59]
Absorbance	miR-155	100 aM–100 fM	100 aM	3-base mismatched miR-155, other DNA	MiRNA detection with citrate-capped Au NPs and PEI capped-Au NPs	[60]
SPR	miR-21 ^(c)^	10 fM–100 pM	3 fM	Blank, miR-141, miR-143	SPR sensor with Au and rGO film that uses DSN for signal amplification	[61]
	let-7b	-	10 fM ^(b)^	Blank, let-7a, let-7c-5p, let-7e		
	miR-15a	5 fM–0.5 nM	0.56 fM (LOQ: 5 fM)	Other DNA sequences	SPRi sensor with isolated Au islands that employs orthogonal signal amplification	[62]
	miR-21, miR-155	10 aM–10 pM ^(a)^ (both)	10 aM (both)	Mismatched miRNA that differs in 1 base (both)	SPR sensor based on two dimensional antimonene nanomaterial and Au nanorods	[63]
LSPR	miR-21	10 pM–100 nM ^(a)^	23–35 fM	miR-16, miR-122, miR-126*, miR-141	Regenerative label-free LSPR sensor based on Au nano prisms	[64]
Silicon Photonic Microring resonators	let-7c-5p	4–250 nM	4 nM ^(b)^	Cross-specificity among the 4 miRNAs, let-7b (only for let-7c-5p)	Label-free miRNA detection in 10 min using arrays of microring resonators	[65]
	miR-21	4–250 nM	4 nM ^(b)^			
	miR-24 ^(d)^	1.95 nM–2 μM	1.95 nM ^(b)^			
	miR-133b	62.5 nM–1 μM	62.5 nM ^(b)^			
	miR-21	20 nM–2 μM	9 nM	Cross-specificity among the 7 miRNAs	Multiplexed miRNA detection via enzymatic signal amplification	[66]
	miR-26a	20 nM–2 μM	4 nM			
	miR-29a	2 nM–2 μM	<1 nM			
	miR-106a	2 nM–2 μM	2 nM			
	miR-222, miR-335	2 nM–2 μM	1 nM			
	miR-16	160 pM–40 nM ^(a)^	160 pM ^(b)^	Cross-specificity among the 4 miRNAs	Microring resonator arrays with amplification using an anti DNA:RNA antibody	[67]
	miR-21, miR-24 ^(d)^, miR-26a	10 pM–40 nM ^(a)^	10 pM ^(b)^			
Interferometry	miR-21, let-7a	1 nM–1 μM (both)	1 nM (both)	miR-122 (miR-21), let-7c-5p (let-7a)	Label-free detection in 15 min with a Mach–Zehnder interferometer (MZI)	[68]
	let-7a	2 nM–20 μM	212 pM	let-7b, let-7c-5p	Optofluidic sensor by assembling a μfiber in lateral contact with a silica capillary	[69]
Surface Enhanced Raman Spectroscopy (SERS)	let-7a, miR-16 miR-133a-3p, (mixtures)	6–150 μM ^(a)^ for all the miRNAs	-	let-7a is detected in a mixture that also contains miR-16, miR-21, miR-24 and miR-133a-3p	Ag nanorod-based SERS for miRNA identification in multicomponent mixtures	[70]
	miR-21	10 fM–100 pM ^(a)^	<10 fM	Blank, a random miRNA	SERS detection of multiple miRNAs using gold and silver nanoprobes and several dyes.	[71]
	miR-31	1 pM–10 nM ^(a)^	1 pM ^(b)^	-		
	miR-141	1 pM–10 nM ^(a)^	<10 fM	-		
	miR-155	1 fM–10 nM	0.67 fM	Blank, miR-21, miR-141, one base mismatched miR-155	SERS combined with DSN amplification using toluidine blue (TB) and CaCO_3_	[72]

^(a)^ In these cases, the dynamic range of the sensor does not follow a linear relationship, or this relationship has not been studied in detail.; ^(b)^ This value corresponds to the lowest concentration detected, but it has not been recognized as the limit of detection (LOD).; ^(c)^ In these sensors, this miRNA is the only one whose LOD and dynamic range was studied in depth.; ^(d)^ In the corresponding articles, this miRNA appears named as miR-24-1. However, the sequence corresponds to hsa-miR-24-3p, also known as hsa-miR-24. The use of the name miR-24-1 can be due to the fact that this miRNA is present in the stem loop sequence hsa-miR-24-1.

**Table 2:** Summary of miRNAs associated with rheumatoid arthritis (RA) mentioned in Section 2.

miRNA	Other Names	Sequence	Ref. (Optical Sensors)	Ref. (RA)
hsa-miR-21-5p	hsa-miR-21	UAGCUUAUCAGACUGAUGUUGA	[37,38,39,40,41,42,43,44,45,46,47,48,49,54,55,56,61,63,64,65,66,67,68,71], [52,70,72] ^(c)^, [59] ^(c),(d)^	[21,23,24]
hsa-let-7a-5p	hsa-let-7a	UGAGGUAGUAGGUUGUAUAGUU	[50,51,52,53,57,58,68,69,70], [39,43,61] ^(c)^	[73,74]
hsa-let-7b-5p ^(a)^	hsa-let-7b	UGAGGUAGUAGGUUGUGUGGUU (2)	[61], [39,43,51,52,53,57,58,65,69] ^(c)^	[75]
hsa-let-7c-5p ^(a),(b)^	-	UGAGGUAGUAGGUUGUAUGGUU (1)	[65], [39,43,50,52,57,58,61,68,69] ^(c)^	[76]
hsa-miR-9-5p	hsa-miR-9	UCUUUGGUUAUCUAGCUGUAUGA	[57]	[77,78]
hsa-miR-15a-5p	hsa-miR-15a	UAGCAGCACAUAAUGGUUUGUG	[62]	[21]
hsa-miR-16-5p	hsa-miR-16	UAGCAGCACGUAAAUAUUGGCG	[56,57,67,70], [64] ^(c)^	[21,22,79]
hsa-miR-24-3p	hsa-miR-24	UGGCUCAGUUCAGCAGGAACAG	[65,67], [70] ^(c)^	[35]
hsa-miR-26a-5p	hsa-miR-26a	UUCAAGUAAUCCAGGAUAGGCU	[66,67], [40] ^(c)^	[35,74]
hsa-miR-29a-3p	hsa-miR-29a	UAGCACCAUCUGAAAUCGGUUA	[66]	[80]
hsa-miR-31-5p	hsa-miR-31	AGGCAAGAUGCUGGCAUAGCU	[56,71]	[22]
hsa-miR-106a-5p	hsa-miR-106a	AAAAGUGCUUACAGUGCAGGUAG	[66]	[81]
hsa-miR-125a-5p	hsa-miR-125a	UCCCUGAGACCCUUUAACCUGUGA	[58]	[35]
hsa-miR-126-3p	hsa-miR-126	UCGUACCGUGAGUAAUAAUGCG	[59] ^(d)^	[35]
hsa-miR-133a-3p	-	UUUGGUCCCCUUCAACCAGCUG	[70]	[82]
hsa-miR-133b	-	UUUGGUCCCCUUCAACCAGCUA	[65]	[35]
hsa-miR-141-3p	hsa-miR-141	UAACACUGUCUGGUAAAGAUGG	[54,71], [38,61,64,72] ^(c)^, [59] ^(c),(d)^	[83]
hsa-miR-155-5p	hsa-miR-155	UUAAUGCUAAUCGUGAUAGGGGUU	[56,60,63,72], [44] ^(c)^	[21,84]
hsa-miR-222-3p	hsa-miR-222	AGCUACAUCUGGCUACUGGGU	[66]	[85]
hsa-miR-335-5p	hsa-miR-335	UCAAGAGCAAUAACGAAAAAUGU	[66]	[86]

^(a)^ These miRNAs are part of the let-7 family and are commonly used in specificity assays where the target miRNA is let-7a. For that reason, in their sequences, the bases in which they differ from let-7a are underlined and the total number of different bases is written between parentheses.; ^(b)^ Let-7c-5p is commonly referred to as let-7c in the articles included in this review. However, based on [36] and after checking that the miRNA sequences were the same, it has been considered more correct to use the name let-7c-5p.; ^(c)^ The miRNA appears in the corresponding article, but used only in a specificity assay.; ^(d)^ The complementary sequence of the corresponding miRNA is used.

**Table 3:** Summary of miRNAs only employed in specificity assays.

miRNA ^(a)^	Other Names	Sequence	Ref (Optical Sensors)
hsa-let-7d-5p ^(b)^	hsa-let-7d	AGAGGUAGUAGGUUGCAUAGUU (2)	[57,58], [59] ^(d)^
hsa-let-7e-5p ^(b)^	hsa-let-7e	UGAGGUAGGAGGUUGUAUAGUU (1)	[38,50,51,53,61]
hsa-let-7f-5p ^(b)^	hsa-let-7f	UGAGGUAGUAGAUUGUAUAGUU (1)	[50,51,53]
hsa-let-7g-5p ^(b)^	hsa-let-7g	UGAGGUAGUAGUUUGUACAGUU (2)	[51,53]
hsa-let-7i-5p ^(b)^	hsa-let-7i	UGAGGUAGUAGUUUGUGCUGUU (4)	[38,51,53]
hsa-miR-122-5p	hsa-miR-122a, hsa-miR-122	UGGAGUGUGACAAUGGUGUUUG	[40,64,68], [59] ^(d)^
hsa-miR-126-5p	hsa-miR-126*	CAUUAUUACUUUUGGUACGCG	[41,64]
hsa-miR-141-5p	hsa-miR-141*	CAUCUUCCAGUACAGUGUUGGA	[40]
hsa-miR-143-3p	hsa-miR-143	UGAGAUGAAGCACUGUAGCUC	[61]
hsa-miR-200b-3p	hsa-miR-200b	UAAUACUGCCUGGUAAUGAUGA	[54]
hsa-miR-210-3p ^(c)^	-	CUGUGCGUGUGACAGCGGCUGA	[45,48,49,55]
hsa-miR-214-3p	hsa-miR-214	ACAGCAGGCACAGACAGGCAGU	[42,45,48,49,55]
hsa-miR-429	-	UAAUACUGUCUGGUAAAACCGU	[54]

^(a)^ If a miRNA is in this table, it does not necessarily mean that it is not connected with RA. It means that, in the articles included in this review, it is only used as a control in specificity assays. For instance, let-7e [22], let-7g [88], miR-143 [89,90], and miR-210 [91,92] (it is not clear if these references mention miR-210-3p or miR-210-5p) are linked with RA. Although both miR-143 and miR-210-3p are detected in [57], they are not studied in depth, so they have not been included in Table 2; ^(b)^ These miRNAs are part of the let-7 family and are commonly used in specificity assays where the target miRNA is let-7a. For that reason, in their sequences, the bases in which they differ from let-7a are underlined and the total number of different bases is written between parentheses.; ^(c)^ MiR-210-3p is commonly referred to as miR-210 in the articles included in this review. However, based on [36] and after checking that the miRNA sequences were the same, it has been considered more correct to use the name miR-210-3p.; ^(d)^ The complementary sequence of the corresponding miRNA is used.

**Table 4:** Optical biosensors for C-reactive protein (CRP) detection.

Optical Technique/Principle	Linear Dynamic Range	LOD	Matrix	Specificity Assays	Comments	Ref.
SPR	2–5 mg/L	1 mg/L	PBS buffer	-	SPR chip with Au surface that uses 2 CRP antibodies for entrapment and detection	[135]
	1.25–80 μg/L ^(a)^	1.2 μg/L (LOQ: 4.6 μg/L)	HBS buffer, diluted human plasma, diluted human serum, diluted human whole blood	HSA, LCN2, HFA, IL-1β, IL-6, IL-8, TNF-α	Au coated SPR chip functionalized with protein A/G	[136]
	10 ng/L–100 μg/L ^(a)^ (with PG in PBS), 10 μg/L–200 μg/L ^(a)^ (with PG in plasma)	10 ng/L (with PG in PBS), 5 μg/L (with PG in plasma)	PBS buffer and diluted human plasma in PBS	Rabbit antigen	SPRi biosensor with Au surface with immobilized Ab without and with protein G	[137]
LSPR	50 μg/L–25 mg/L (PBS)	50 μg/L (PBS)	PBS buffer and diluted blood serum (10 times) in PBS	-	Label-free sensor that measures the OD change with 2 antibodies for capture and detection	[138]
	50 μg/L–3 mg/L ^(a)^ (buffer)	~50 μg/L (buffer)	Tris-HCl modified buffer and 1% diluted human serum in buffer	HSA	LSPR sensor based on Au NPs on which PMPC was grafted using ATR polymerization	[139]
	10 μg/L–10 mg/L	11.28 μg/L	PBS buffer	Hb, TF and HSA (separately and in mixture)	Cuvette cell system that uses Au NPs and a substrate modified with APTES	[140]
	100 fg/L–1 mg/L	100 fg/L	Tris-HCl buffer	-	LSPR biosensor based on nanostructured AAO substrates with Au NP labelled Ab	[141]
LMR	62.5 µg/L–1 mg/L ^(a)^	62.5 µg/L	TBS buffer	Urea and creatinine	LMR sensor with ITO film using the layer by layer (LbL) technique	[142]
Refractive index change	100 μg/L–10 mg/L ^(a)^	100 μg/L ^(b)^	Diluted human serum (10 times) in PBS buffer	-	Label-free metal clad leaky waveguide (MCLW) sensor with nitrocellulose	[143]
Etched Fiber Bragg gratings (eFBG)	10 μg/L–100 mg/L	10 µg/L	Deionized water	Urea, glucose, and creatinine	Graphene oxide (GO) coated eFBG sensor	[144]
	0.8 pg/L–1.2 µg/L ^(c)^ (buffer)	0.82 pg/L (buffer), 27.6 pg/L (plasma)	Modified aptamer buffer and diluted CRP deficient human plasma	Urea and ascorbic acid	Gratings fabricated using a femtosecond pulsed laser and etching done with hydrofluoric acid	[145]
Reflectometric interference spectroscopy (RIfS)	50–400 µg/L	63.8 µg/L	HBS-P buffer	BSA, HSA	RIfS based sensor with two TiO_2_ layers prepared by liquid phase deposition (LPD), sensitive layer includes anti-CRP and PL	[146]
Colorimetry	1 μg/L–10 mg/L (DI water)	1 µg/L (DI water)	Deionized water and human serum spiked with CRP	-	Swarm biosensing platform based on the plasmonic signal from Au NPs sensors.	[147]
Photoluminescence	75 ng/L–1.65 mg/L (diluted PBS)	45 ng/L (diluted PBS)	100 times diluted PBS and human serum spiked with CRP	GA, thrombin, TF, TNF-α used as control proteins	Nanosensor based on DNA aptamer attached to a QD and a Au NP	[148]
Fluorescence	1–300 mg/L (buffer)	0.3 mg/L (buffer)	Tris buffer, human serum spiked with CRP	-	Lateral flow immunoassay based on double Ab sandwich technique using CdTe QDs	[149]
	20 pg/L–12.5 ng/L (PBS)	20 pg/L (PBS)	PBS buffer and human serum	Albumin	Label-free biochip based on MSF that alters fluorescence of FAI using its ligand PEA	[150]

^(a)^ In these cases, the dynamic range of the sensor does not follow a linear relationship, or this relationship has not been studied in detail.; ^(b)^ This value corresponds to the lowest CRP concentration detected, but it has not been recognized as the LOD.; ^(c)^ Particular case, the dynamic range follows the Langmuir–Freundlich isotherm model.

**Table 5:** Optical biosensors for detection of other RA biomarkers.

Biomarker	Optical Technique/Principle	Linear Dynamic Range	LOD	Matrix	Comments	Ref.
RF	Chemiluminescence	5.3–485 IU/mL ^(a)^	5.3 IU/mL ^(b)^	Human sera (1:10 dilution in modified PBST)	Screen printed microarray, immobilization strategy based on an aniline derivative	[168]
ACPA	SPR imaging (SPRi)	-	0.5 pM ^(b)^	Huma sera (1:50 dilution in PBS) from 50 RA patients and 29 controls)	Label-free sensor based on SPR dip angle scanning	[169]
		-	-	Human sera (1:50 dilution in PBS) from 374 early RA patients	SPRi analysis in a sensor chip with gold surface consisting of a 48 spot microarray	[170]
IL-6	Fluorescence	1 pg/mL–1 ng/mL (buffer)	0.9 pg/mL (buffer)	Tris buffer, human serum spiked with IL-6	Lateral flow immunoassay based on double Ab sandwich technique using CdTe QDs	[149]
Histidine	Fluorescence	500 nM–100 μM	76 nM	PBS buffer	Fluorescence sensor based on CuAAC, a type of click reaction.	[171]
		1 nM–5 μM	0.6 nM	Human plasma (diluted with citrate solution PBS and acetonitrile)	Optical sensor that uses Eu-Norfloxacine complex doped in a sol-gel matrix	[172]

^(a)^ In these cases, the dynamic range of the sensor does not follow a linear relationship, or this relationship has not been studied in detail; ^(b)^ This value corresponds to the lowest concentration detected of the corresponding biomarker, but it has not been recognized as the LOD.
